# Discovery and Development of Voxvoganan: First-in-Class Synthetic Antimicrobial Peptidomimetic

**DOI:** 10.1021/acs.jmedchem.6c00102

**Published:** 2026-04-20

**Authors:** Wenche Stensen, Dina Jurman, Jon Lind, Pontus Lundberg, John S. M. Svendsen

**Affiliations:** † UiT The Arctic University of Norway, 9019 Tromso̷, Norway; ‡ Amicoat AS, Mo̷lnholtet 42, 9414 Harstad, Norway; § 694346 Amicoat AS, Hagalo̷kkveien 26, 1383 Asker, Norway; ∥ Pharma Holdings AS, Sjo̷gata 2, 9008 Tromso̷, Norway

## Abstract

Voxvoganan is a synthetic antimicrobial peptidomimetic designed by applying the principles of natural antimicrobial peptides sharing many of the attractive properties, such as a wide antimicrobial spectrum and fast antimicrobial action. In contrast to the natural peptides that typically consist of 15–25 residues, voxvoganan contains only three amino acids, comparable to a classical small molecule drug, and can be manufactured on an industrial scale using solution phase methods. Voxvoganan has been developed both as a drug for the early treatment of respiratory viral infection and as an antimicrobial active pharmaceutical ingredient in medical devices to avoid microbial fouling, thus reducing the infection risk connected with the use of these devices. During development, voxvoganan has been through toxicology and safety studies as well as Phase 1 and 2 clinical studies. When voxvoganan hits the market as a first-in-class drug, the unique potential of antimicrobial peptides will finally reach patients.

## Graphical abstract

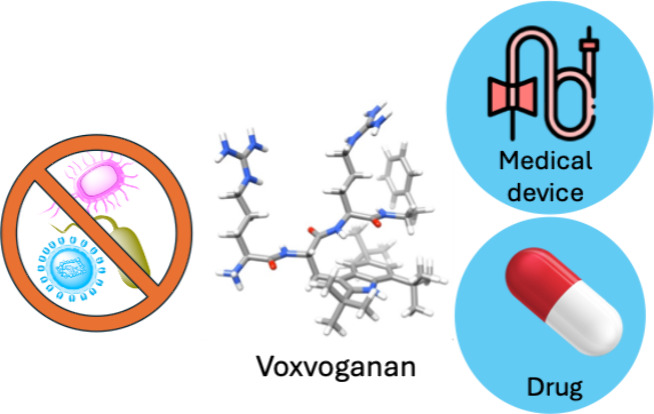

## Introduction

Antimicrobial peptides (AMPs) play an important role in the ancient host defense system and can trace their origins to more than 500 million years ago.^1^ The importance of antimicrobial peptides to our health is obvious, evidenced not only by their long evolutionary history but also by their widespread presence across the phyla in the tree of life. As a group, the AMPs are diverse from a molecular viewpoint.^2,3^ Some common themes are prevalent. The peptides are relatively short (ranging from 12–50 amino acids), and many AMPs contain an abundance of specific amino acids such as the proline-rich and tryptophan/arginine-rich peptides. AMPs are also distinguished from other antimicrobial matter by their broad spectrum of activity, fast microcidal action, and low propensity for resistance development. Furthermore, many AMPs also have immunomodulatory properties as an important part of their function.^4^

These favorable properties have resulted in AMPs being hailed as the antibiotics of the future, although the natural AMPs have a multitude of inherent challenges that must be resolved before an AMP can be used as a clinical drug. The mode of action of AMPs is to damage the cell membrane of procaryotes causing lysis, thus opening the potential of affecting the eukaryote cell membranes as well, thus causing cytotoxic and hemolytic side effects. Many AMPs have reduced activity in the presence of host factors, such as ions and blood serum, and most AMPs are easily hydrolyzed by proteases. Finally, industrial production of long AMPs requires lengthy solid phase methods with high associated costs, hence precluding inexpensive commercial manufacture. A drug-like AMP must at least have high antimicrobial activity, low toxicity to mammalian membranes, high protease and environmental stability, good serum stability, activity in the presence of host factors, and the ability to be produced at GMP-quality at a reasonable cost. In addition to these rather obvious requirements, AMPs must also adhere to all other properties required for a viable commercial drug, such as favorable drug metabolism, pharmacokinetic (DMPK) properties, and toxicology and safety qualities.

## Results

We started the creation of a viable AMP drug candidate more than three decades ago by studying an antimicrobial peptide fragment derived by peptic degradation of lactoferrin, lactoferricin.^5^ These studies, which have been reviewed elsewhere,^6^ led us to the idea that cationic charge and tryptophan residues were the major factors determining antimicrobial efficacy. It was surprisingly found that the 25-residue bovine lactoferricin peptide could be shortened to peptides with just 5 residues containing only Trp (W) and Arg (R) residues while still retaining good antibacterial activity.^7^

### Pharmacophore

The discovery that very short AMPs could contain just cationic residues and a certain number of Trp residues prompted the preparation of a library of short peptides containing only Arg and Trp residues. The intention of this library was to define an AMP pharmacophore, i.e., the shortest RW-peptide possible with good antimicrobial activity against the selected microorganism. The library was further restricted to only containing balanced peptides; i.e., peptides with an even number of amino acids should contain the same number of Arg- and Trp-residues, and odd numbered sequences would allow for one type of amino acid more than the other type. Furthermore, the peptides in the library should have an amidated C-terminus to avoid any adverse charge effect from an anionic C-terminal carboxylic acid. The library was tested against a *Staphylococcus aureus* and an *Escherichia coli* strain, and a selection of the results is presented in Table 1.

**Table 1:** Physical Properties in the Form of Number of Cationic Charges (N-Terminus and Side Chains) and Lipophilic Bulk Represented by Number of Trp-Residues versus MIC (mg/L) against *E. coli* and *S. aureus* for Peptide Amides^a^

	number of sites		MIC	
peptide sequence^b^	cationic	bulk	*E. coli*	*S. aureus*
WRWRWR-NH_2_	4	3	10	7.5
RWRWRW-NH_2_	4	3	5	5
RRRWWW-NH_2_	4	3	5	5
RWWWRR-NH_2_	4	3	25	5
WWRRRW-NH_2_	4	3	25	10
				
WRWRW-NH_2_	3	2	15	10
RWRWR-NH_2_	4	3	200	25
				
WRWR-NH_2_	3	2	>200	200
WRRW-NH_2_	3	2	>200	200
RWWR-NH_2_	3	2	>200	100
				
WRW-NH_2_	2	2	>200	100
RWR-NH_2_	3	1	>200	>200

a One letter code according to IUPAC IUB: Arg = R, Trp = W.

b Data collected from Stro̷m et al.^8^ *Escherichia coli* ATCC 25922, *Staphylococcus aureus* ATCC 25923.

The physical properties of the peptides were expressed in terms of the number of positive charges (C) and lipophilic bulk as the number of side chains of the size of an indole (B). The lower limit of efficacy was set as a MIC value of 100 mg/L. Interpretation of Table 1 leads to the definition of a pharmacophore for RW-peptides as 3B + 3C for the Gram-negative *E. coli* and just 2B + 3C for the Gram-positive *S. aureus*. An interesting observation in Table 1 is that the sequence of the amino acid is of only minor importance for the antimicrobial efficacy compared to the number of B and C. Additional experiments confirmed the definition of the pharmacophores to 2B + 2C for *S. aureus* and 3B + 2C for *E. coli*. Furthermore, the lipophilic bulk is not limited to using an indole since an indole can be substituted by a benzyl group with a minimal loss of activity, and the charged side chain is not restricted to arginine as lysine could also be substituted, albeit with some loss in antimicrobial efficacy. Finally, both the exact sequence of the amino acid and the stereochemistry of the individual amino acids in the peptides played only a minor role in determining antimicrobial efficacy.^8^ These last observations are particularly revealing, as they strongly point toward a nonselective receptor like the cell membrane.

### The Importance of Lipophilic Bulk

The importance of the cationic residues is readily understood in light of the interaction between the AMPs and the microorganism’s anionic outer leaflet of the membrane. However, the importance of the tryptophan residue could not be explained so easily. To determine why tryptophan played this role, an experiment was performed where Trp in position 6 or position 8 in the LFB sequence (Phe-Lys-Cys-Arg-Arg-**Trp**_**6**_-Gln-**Trp**_**8**_-Arg-Met-Lys-Lys-Leu-Gly-Ala) was replaced with a series of lipophilic residues (X) with different physical characteristics as shown in Figure 1.^9^ The amino acids selected for substitution included Phe with a smaller lipophilic and aromatic side chain. Then a set of isosteric substitutions followed, such as benzothiazolalanine (Bal) without the NH-group of indole, and the two isomers of the fully aromatic and lipophilic amino acid naphthylalanine (1-Nal and 2-Nal) were selected to explore the importance of hydrogen bonding ability, polarity, and the indole geometry of Trp. The antibacterial activity of the LFB-peptides with these amino acids showed that neither the hydrogen bonding ability nor the amphipathicity of the indole system in tryptophan were essential properties for the antibacterial activity of the peptides. Finally, the Trp residues were replaced with residues containing larger aromatic hydrocarbon side chains with two phenyl groups, β,β-diphenylalanine (Dip) with increased 3-dimensional bulk and biphenylalanine (Bip) with an elongated shape and an anthracenyl system (anthracenylalanine (Ath)). The antibacterial activity of the peptides containing the substitutions is compared to the native LFB-sequence and compiled in Table 2. Inspection of Table 2 reveals that the Trp indole NH-group was not responsible for the importance given that replacing the indole with isosteric naphthyl groups lacking the NH-moiety hardly changed the antimicrobial activity. Steric effects appeared to play a small role as the 2-Nal peptides were more active against bacteria than the 1-Nal analogs. However, increasing the bulk and lipophilicity of the side chains yielded more active peptides. The hypothesis for this observation is that aromatic and lipophilic hydrocarbon residues may be able to penetrate more deeply into the bacterial cell membrane, making the AMP more efficient in disrupting the bacterial cell membrane. The results point toward the size, shape, and aromatic character of Trp being the most important features for the activity. Interestingly, the most active peptides against *E. coli* had broad Ath or 3-dimensional Dip residues, while *S. aureus* was more susceptible to the longer and needle shaped Bip residue. Importantly, the Trp residues could be replaced with other even larger aromatic nongenetically encoded residues like Tbt, accompanied by a general gain in antibacterial efficacy irrespective of the Gram-type as a result.

**Figure 1:**
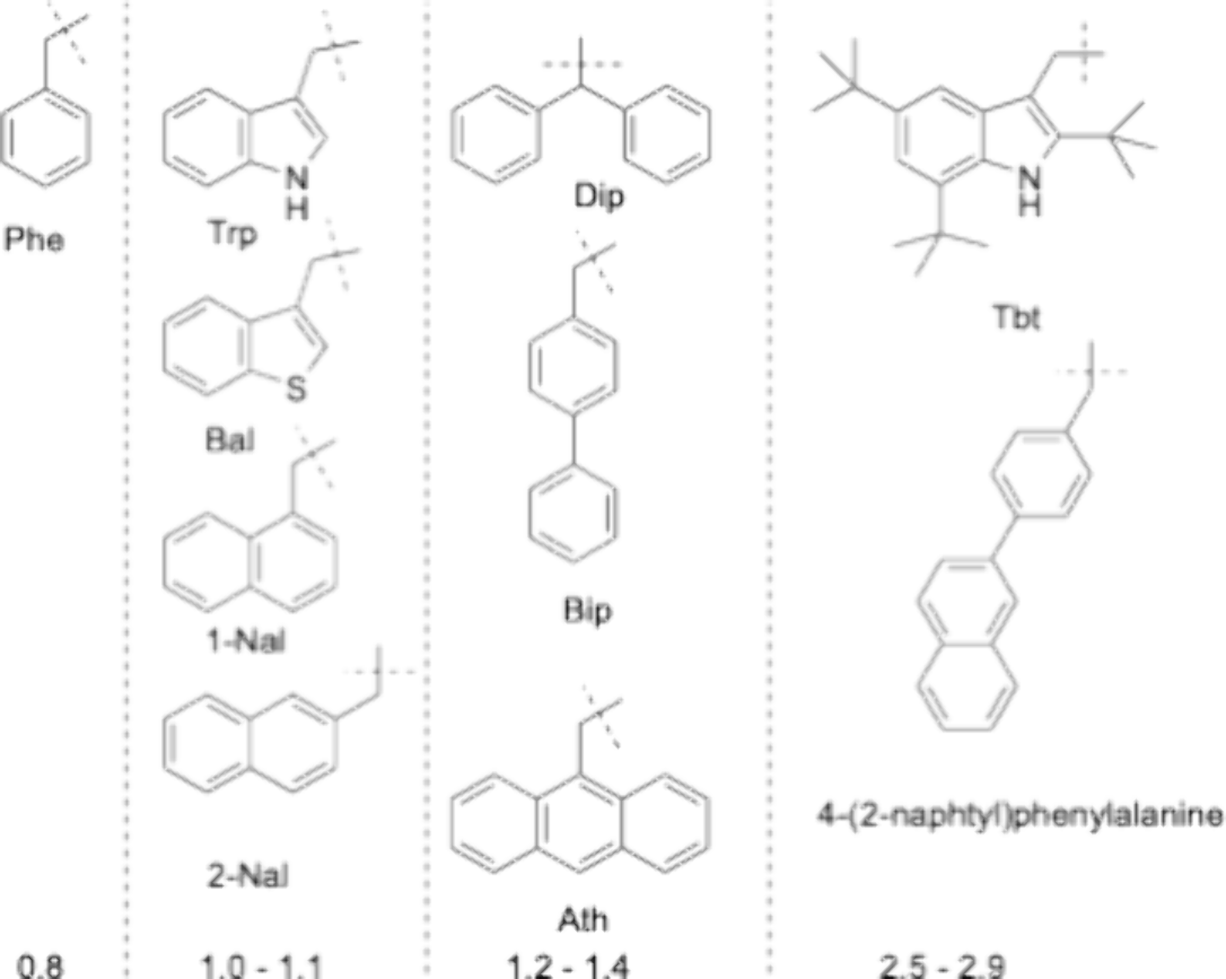
Structure of the side chain with calculated size relative to tryptophan of aromatic amino acid X substituted in position 6 or 8 in the LFB sequence.^6^

**Table 2:** Minimum Inhibitory Concentration (MIC) in mg/L against *E. coli* and *S. aureus* of LFB Peptides Where the Trp Residue in Position 6 or 8 Is Substituted with a Bulky and Lipophilic Residue^a^

		Position 6		Position 8	
bulky amino acid	side chain volume relative to Trp^10^	*E. coli*	*S. aureus*	*E. coli*	*S. aureus*
Trp		50	100	50	100
Phe^9^	0.77	50	>300	25	>300
Bal^9^	1.05	15	75	15	35
1-Nal^9^	1.12	50	150	20	100
2-Nal^9^	1.12	20	75	10	50
Dip^10^	1.33	7.5	35	7.5	35
Bip^10^	1.33	25	15	15	15
Ath^10^	1.43	12.5	35	10	15
Tbt^10^	2.52	12.5	10	12.5	5

a *Escherichia coli* ATCC 25922, *Staphylococcus aureus* ATCC 25923.

### The Creation of SAMPs – Synthetic Antimicrobial Peptidomimetics

With the pharmacophore model unraveled and the newfound insight into the role of the tryptophan residues in AMP’s, the next step was to combine these two lines of research and create a small library of effective synthetic supershort antimicrobial peptides with the sequence X-Arg-Y where amino acid X was selected from the residues depicted in Figure 1, and Y was varied between small groups such as an amide or a methyl ester and large group such as 4-phenylbenzylester.^11^ Analysis of the antibacterial efficacy of the resulting peptides as a function of the lipophilicity and bulk of X and Y would be used to define B.

The study revealed that a peculiar, nearly arithmetic relationship between the B-units exists, corroborating both the applicability and the nature of the pharmacophores. The required 2 B-units can be evenly distributed with one in the side chain of the lipophilic amino acid and one in the C-terminal modification or alternatively can be concentrated in the C-terminal modification or combined in a superbulky lipophilic amino acid side chain. If the combined size of the B-units is constant, then the antibacterial activity of the peptides is hardly affected by how the B-groups are distributed. The largest superbulky residue was 2,5,7-tri-*tert*-butyltryptophane (Tbt) with a side chain volume approximately 2.5 times larger than the largest genetically encoded residue, tryptophan. The peptides containing a Tbt residue were the most active against both *S. aureus* and *E. coli* in the series. The pharmacophores are thus robust and represent a new class of AMPs, synthetic antimicrobial peptidomimetics (SAMPs).

The antibacterial results obtained with peptides in this library verified that very effective antibacterial peptides could be prepared utilizing this concept with the dipeptide Tbt-Arg-NHBzl having MIC values of 10 mg/L against *E. coli* and 2.5 mg/L against *S. aureus* as a premier example. Such an excellent antimicrobial efficacy of a dipeptide was unheard of, leading to concerns that the medicinal chemistry used to refine the AMPs into viable SAMP drug candidates might have shifted from the microbe selective natural AMPs into the realm of nonselective disinfectants with an unacceptable eukaryotic cytotoxicity.

### Peptidase Stability

One required property for a viable peptide drug candidate is a sufficient stability toward degradation at the site of action. Enzymatic hydrolysis into shorter peptides or amino acids is probably the most important biological degradation mechanism for peptides including AMPs. To investigate the stability against enzyme catalyzed hydrolysis of SAMPs, a library of tripeptides all adhering to the anti-staphylococcal pharmacophore, with the sequence Arg-X-Arg-NHY, where X is selected among the residues shown in Figure 1 and Y is a lipophilic C-terminal modification, was prepared. As expected from the pharmacophore model, the majority of the peptides in the series had good to excellent antibacterial activity against Staphylococci. Trypsin was chosen as the model enzyme for hydrolysis of the cationic antimicrobial peptides in the library due to its relevance and a hydrolytic specificity matching the peptides.^12^ Trypsin thus cleaves peptides at the C-terminal side of positively charged residues, such as Arg and Lys, either forming dipeptides due to hydrolysis of the N-terminal cationic amino acid, forming an X-Arg-NHY dipeptide, or tripeptide carboxylic acids, Arg-X-Arg-OH, due to cleavage of the C-terminal modification. Both hydrolysis products have one positive charge less than the original peptides (provided that the tripeptide carboxylic acid is deprotonated to the carboxylate at the actual pH) and are thus expected to be significantly less active against microbes than their starting peptides.

The hydrolytic stability observed revealed that the tripeptides with bulky X-residues were surprisingly good candidates for tryptic degradation with relatively short half-lives. This finding was not anticipated as trypsin has a pronounced endopeptidase specificity, being most active on peptides containing 6 amino acids or more. However, when X is a β,β-disubstituted superbulky residue, trypsin was unable to catalyze a hydrolysis. A finding in the experiment was the unexpected stability of peptides not containing superbulky residues but with a certain C-terminal modification. The tripeptides with phenylethyl amide showed excellent hydrolytic stability, whereas the corresponding peptides with benzyl amide and phenylpropyl amide (with a methylene group less or a methylene group more in the side chain, respectively) both had much lower hydrolytic stability.

### The Lead Series

When the hydrolytic stability results were combined with the SAMP-pharmacophore, a lead series library could be designed using the following simple features: the peptides should contain three amino acids, two arginine residues flanking one lipophilic superbulky Tbt-residue or 4-(2-naphtyl)­phenylalanine. Furthermore, the peptides should have a lipophilic C-terminal capping group that encompasses good stability. Adhering to the SAMP-pharmacophore should ensure the good expected antimicrobial efficacy. However, unacceptably high levels of cytotoxicity toward eukaryotic cells had to be avoided. The C-terminal capping group was a methyl ester, isopropyl amide, or phenethyl amide. A library adhering to these principles was thus designed and contained the following peptides, **105**, **107**, **108**, **109**, and **110**, with structures outlined in Scheme 1. Four of the five peptides were designed around the superbulky Tbt residue, while **107** contains the 4-(2-naphtyl)­phenylalanine instead. Otherwise, the series varied in the C-terminal modification **108** having a simple methyl ester, **105** having an isopropyl amide modification, **107** and **109** are both phenylethyl amides, and **110** is a *n*-hexylamide.

**Scheme 1:**
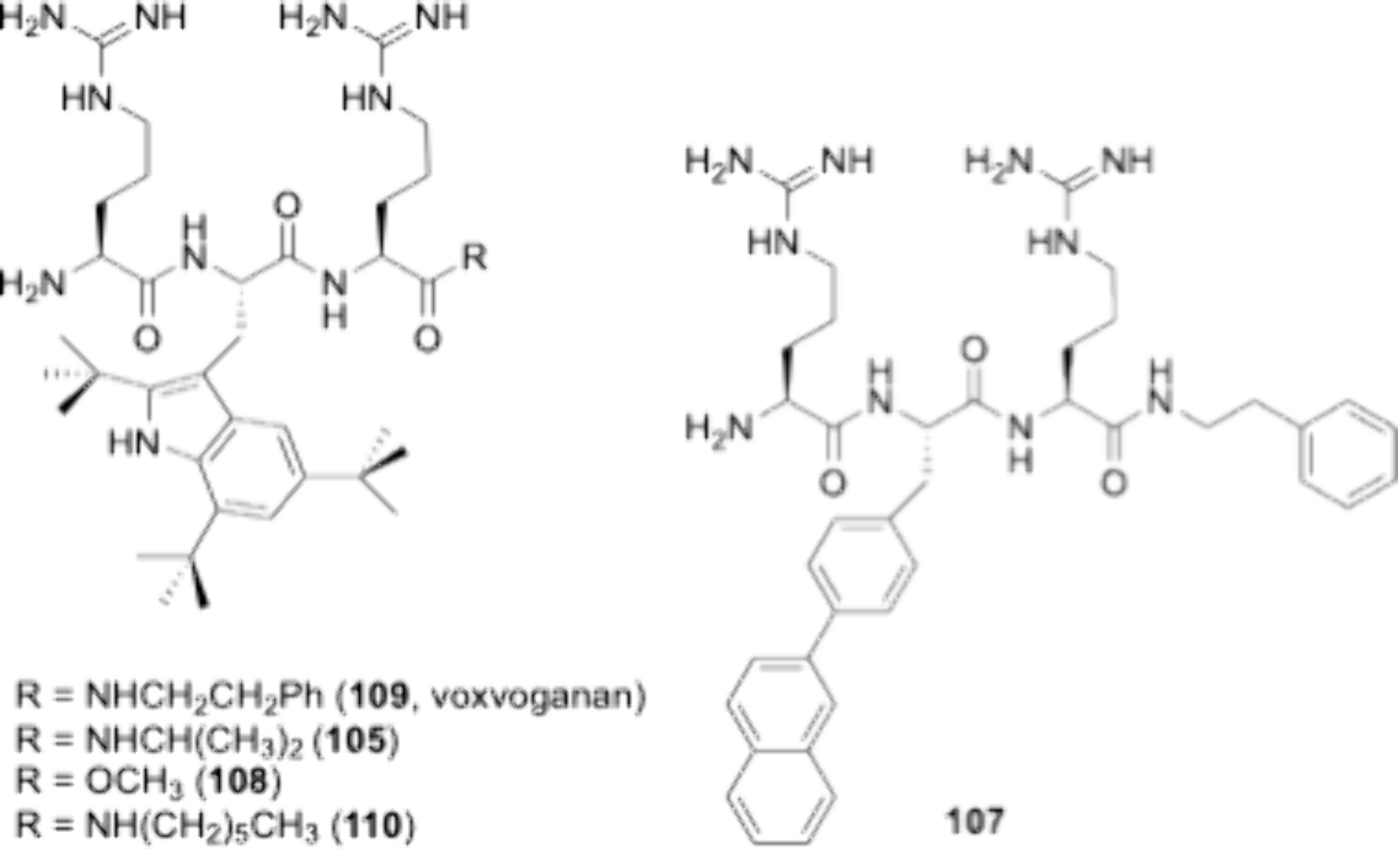
Peptides in the Lead Series^a^

a Peptides **105**, **108**, **109**, and **110** all have Tbt as the lipophilic and bulky residue with varying C-terminal modifications, whereas peptide **107** is derived from 4-(2-naphtyl)­phenylalanine. All amino acids have l-absolute configuration.

The peptides in the lead series were first evaluated with regard to antimicrobial efficacy, measured as MIC against a wide panel of microorganisms including *S. aureus*, MRSA, *Streptococcus pyogenes*, *E. coli*, *Pseudomonas aeruginosa*, *Candida albicans*, and *Aspergillus niger*. The results are listed in Table 3.

**Table 3:** Antimicrobial Efficacy Measured as MIC in Milligrams Per Liter of a Series of AMPs against a Set of Microorganisms^a^

	Gram positive			Gram negative		fungi	
peptide	*S. aureus*	MRSA	*S. pyogenes*	*E. coli*	*P. aeruginosa*	*C. albicans*	*A. niger*
**105**	8	8	16	32	32	32–64	32
**107**	4	4	32	32	16	2	4
**108**	8	8	32	64	32	32	16
**109**	4	4	16	8	8	8	8
**110**	4	4	8	4–8	4–8	4–8	4

a *Staphylococcus aureus* ATCC 25923, MRSA SSCmec Type 1, *Streptococcus pyogenes* Macrolide (MLS) resistant clinical isolate, *Escherichia coli* ATCC 25922, *Pseudomonas aeruginosa* ATCC 27853, *Candida albicans* ATCC90028, *Aspergillus niger* GR Micro collection.

The peptides **107**, **109**, and **110** excelled in the series, with good activity against all tested microorganisms, Gram+ and Gram– bacteria, as well as the yeast and the filamentous fungus. Peptide **110** had the broadest and most uniform activity against the complete microorganism library with MIC values in a narrow interval between 2 and 8 mg/L. Peptides **105** and **108** on the other hand had significantly weaker antimicrobial efficacy than the rest of the lead series.

The antimicrobial action of AMPs proceeds through mechanisms involving membrane lysis. The practical utility of the peptides is thus dependent on their ability to lyse the prokaryotic (or fungal) cell membrane at concentrations at which mammalian membranes are left unharmed. The fear was that the refinement of size and composition of the peptides toward optimal antimicrobial activity creating the smallest possible SAMP had moved the natural AMPs toward being a nonselective general membrane lysing disinfectant. An eukaryotic cytotoxicity assay was thus employed as a counter screen against antimicrobial efficacy to assess the antimicrobial selectivity. The peptides in the lead series were tested for their hemolytic activities using erythrocytes and for general eukaryotic cytotoxicity against MRC 50 fibroblasts. The data from the cytotoxicity screen compiled in Table 4 showed that the peptides in the lead series were cytotoxic at levels similar to the levels seen for clinical drugs like daptomycin and mafenide. On the other hand, there is a correlation between antibacterial MIC and membranolytic activity on eukaryotic cells as the two peptides with the least lytic activity against the microorganisms, **105** and **108**, were also the least cytotoxic. These two peptides were thus excluded from further development due to their insufficient general antimicrobial efficacy. Among the peptides with pronounced and broad activity, peptide **110** was the most cytotoxic followed by **107** and **109**. Peptide **110** thus fell out of the lead series due to unacceptable cytotoxicity. Comparing MIC values with IC_50_ values directly is not possible. The MIC determination is performed on living and multiplying cells in the log phase in a nutritious growth medium, while the IC_50_ determination is performed in a nutrient-poor buffer to make the assay as sensitive as possible to detect even quite low cytotoxic activity. Hence, comparing trends between MIC and IC_50_ provides valuable insights, a direct numeric comparison overestimates the potential for cytotoxic effects.

**Table 4:** Lytic Activity (IC_50_ in mg/L) against Erythrocytes and MRC 50 Fibroblasts, and Retention Time on a Reverse Phase (RP) HPLC of Peptides in the Lead Series^a^

peptide	hemolysis (EC_50_)	fibroblast (IC_50_)	retention time (min)
**105**	720	368	17.43
**107**	200	48	15.74
**108**	>1000	270	17.02
**109**	60	77	19.42
**110**	32	58	20.83

a Gradient: (75:25 water/acetonitrile both containing 0.1% TFA) (3 min) – linear increase to 40:60 in 19 min – rest of time in 40:60). For further details see the Materials and methods section.

An interesting observation is that the cytotoxicity of the peptides closely followed the RP HPLC retention time. The longer the retention time, the more toxic the peptides became. It is well-known that the lipophilicity of the peptides correlates with the retention time on RP HPLC,^13^ and thus the lipophilicity can be estimated using retention time. In the Tbt-derived peptides, lipophilicity increases with increased numbers of carbon atoms in the C-terminal modification, an effect that is expected, as the C-terminus is the only variable in the series. There is a clear correlation between the lipophilicity of the Tbt-series peptides and their eukaryotic cytotoxicity. The correlation between the toxicity and the lipophilicity of the peptides indicates that the interaction of the peptides with the eukaryotic membranes is mainly of a lipophilic nature, in contrast to the interaction between the peptides and the prokaryotic that is driven by initial charge interactions.

The 4-(2-naphtyl)­phenylalanine peptide **107** has a significantly shorter retention time than the corresponding Tbt-based peptide **109**, and hence peptide **107** appears to be much less lipophilic than **109** despite similar numbers of heavy atoms in the side chain. This difference in apparent lipophilicity in **107** and **109** is not reflected in their similar eukaryotic toxicity. An explanation can be sought in the restricted “stiff” backbone conformation of the Tbt-peptide **109** ^14^ compared with the much more freely rotating backbone conformation made possible by the sterically less encumbered 4-(2-naphtyl)­phenylalanine side chain of peptide **107**. The net effect of the backbone stiffness is that the Tbt peptides like **109** become more amphipathic, thus forcing the lipophilic groups to interact with the lipophilic column surface resulting in long retention times, whereas **107** is less conformationally restricted in the backbone and therefore less amphipathic, ending up with a shorter retention time and an apparent low lipophilicity compared to its Tbt-containing counterpart. However, the more flexible backbone of **107** still allows the peptide to assert conformations where the peptide interacts well with eukaryotic membranes; hence, the eukaryotic cytotoxicity is at least at the same level as the Tbt-derived peptides. Peptide **109** is thus restricted to always being in an “active” conformation dictated by the relative stereochemistry of the Tbt and the C-terminal Arg residue (both L),^14^ whereas peptide **107** adopts a conformation that maximizes membrane interaction, a conformation that may be different in prokaryotic and eukaryotic membranes.

Hydrolytic stability was also included in the criteria used for ranking of the peptides. As shown in Table 5, the peptides containing Tbt were stable in the presence of trypsin, whereas **107** was slowly degraded, however at a rate that could not exclude the compound based on hydrolytic stability. Peptide **109** was found to be stable in human blood plasma; hence, there were no effective pathways for degrading the peptide during circulation. This finding does of course not preclude any effect on the peptide provided by distribution, metabolism, and excretion in a complete organism. The totality of these effects was found by determining the *in vivo* *t*_1/2_ of peptide **109** post a single dose of 15 mg/kg intravenous (iv) or 30 mg/kg subcutaneous (sc) administration in mice (Figure 2).

**Figure 2:**
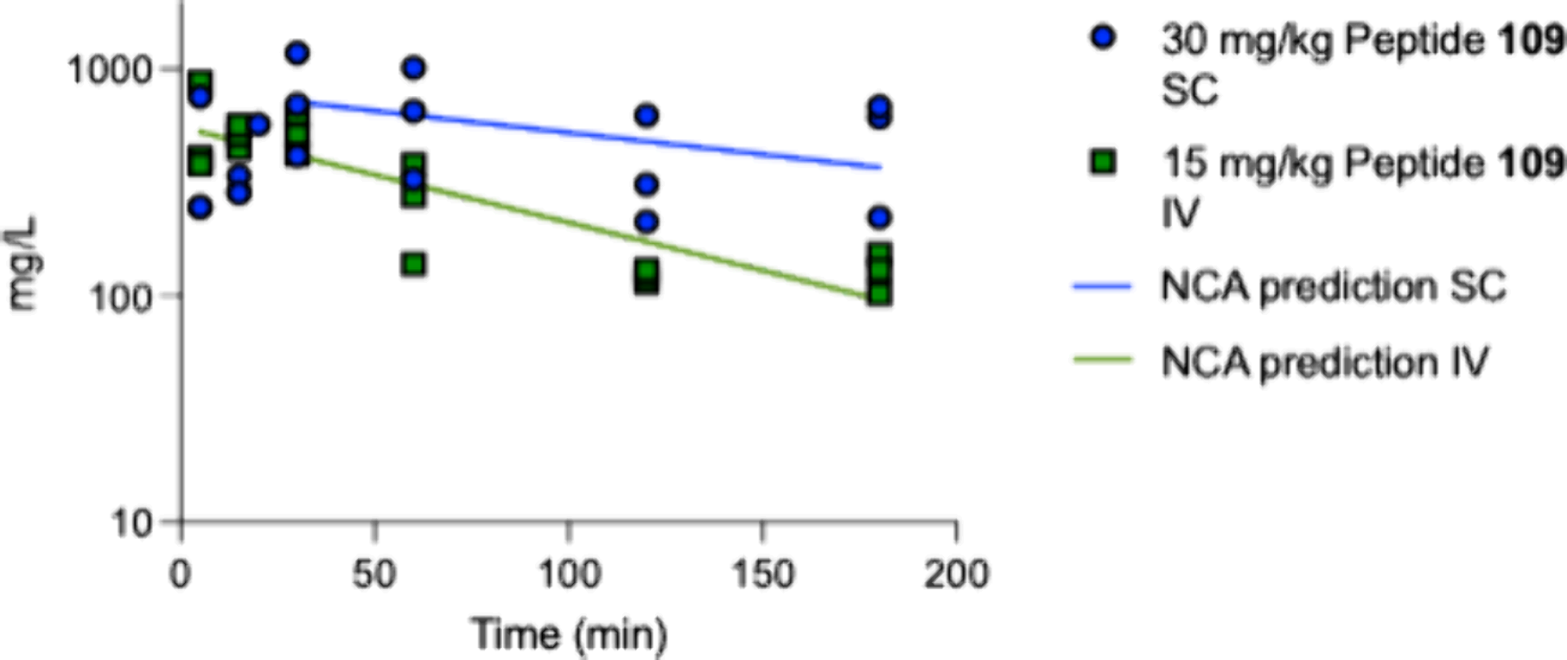
Concentration of peptide **109** on the blood plasma as a function of time after a subcutaneous (SC) administration of 30 mg/kg or an intravenous (IV) administration of 15 mg/kg of peptide **109**. Best-fit predictions using a noncompartmental analysis (NCA) are also given as straight lines.

**Table 5:** Half-Life (*t*_1/2_, h) in Blood and Metabolism of Peptides **107** and **109**

	in vivo (mice)		in vitro	
peptide	*t*_1/2_ (min)		*t*_1/2_ in trypsin	*t*_1/2_ in plasma
	iv	sc		
**107**			30 h	
**109**	71	156	stable	stable

The *t*_1/2_ in blood was estimated to be 716 min after a bolus injection in the tail vein and 156 min after a subcutaneous injection (Table 5).

The short half-lives observed could limit the systemic utility of the peptide. Further studies showed that the peptide was rapidly taken up by the liver and excreted unmetabolized in the feces and urine.^15^

### The Selection of Peptide **109** as the Drug Candidate

At this stage in the development cycle, it was clear that the important properties of peptides **107** and **109** were in total better than the rest of the lead group; on the other hand, the properties of **107** and **109** were so similar that the selection between the compounds could be arbitrary. The final selection of peptide **109** rather than **107** was in the end based on an assessment of the industrial manufacturing process that appeared to be simpler for the Tbt amino acid in peptide **109** than the 4-(2-naphtyl)­phenylalanine amino acid in peptide **107**. The Tbt-amino acid in **109** can be prepared in a single step from l-Trp by exhaustive Friedel–Crafts *tert*-butylation (Scheme 2).^16^ The 4-(2-naphtyl)­phenylalanine amino acid is prepared by a Pd-catalyzed coupling of l-4-iodophenylalanine with 2-bromonapthalene. This process turned out not to be as scalable as the Friedel–Crafts reaction and required expensive starting materials and catalysts.

**Scheme 2:**
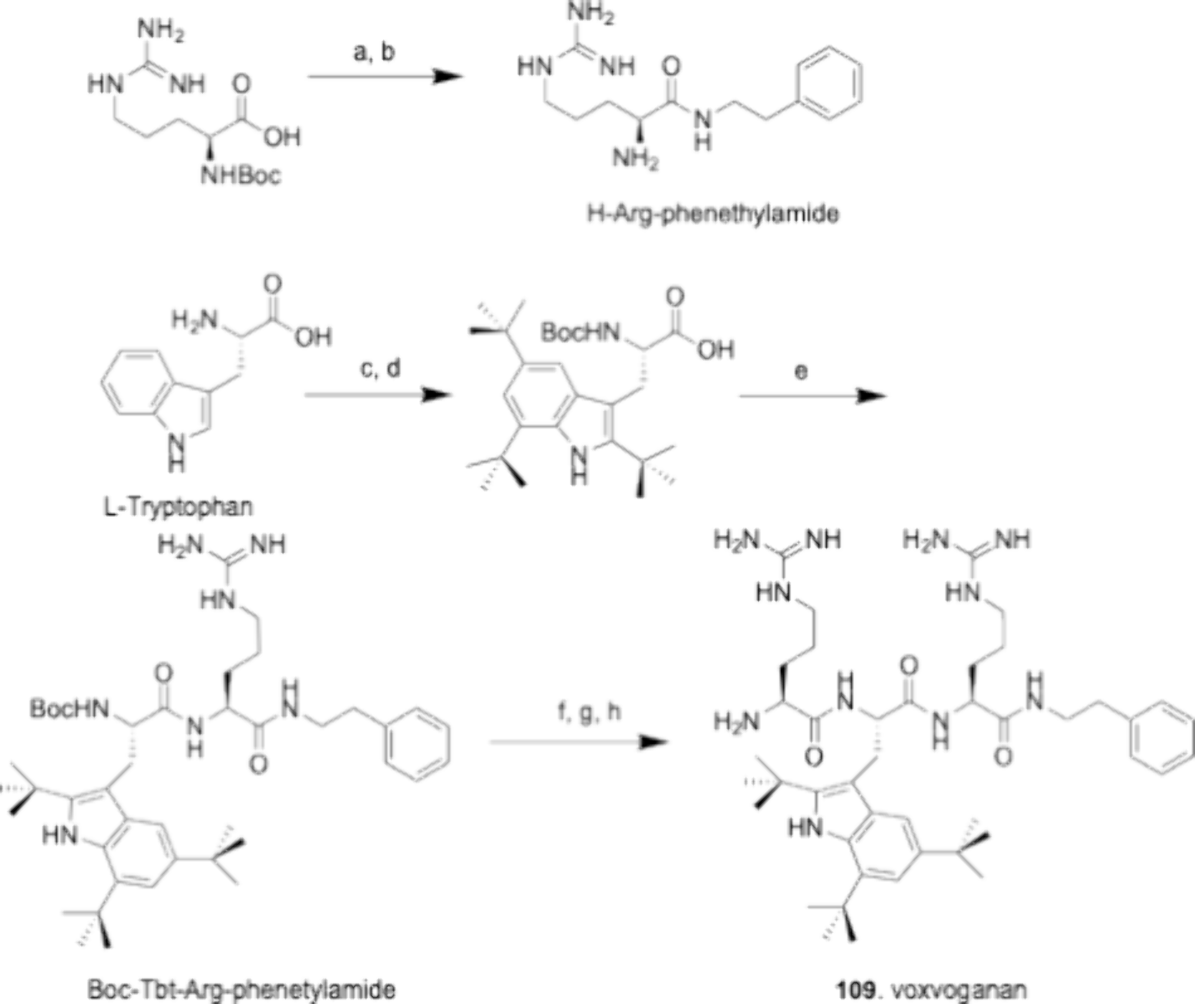
A Laboratory Scale Process for the Preparation of Peptide **109** Using Boc-Protected Amino Acids and HBTU/HOBt Couplings^a^

a Reagents and conditions: (a) Phenethyamine, HBTU, HOBt, DIPEA, DMF, (b) TFA, precipitation in diethyl ether, (c) t-BuOH, TFA, 70 °C, 4 h, crystallization, (d) di-*ter*t-butyldicarbonate, dioxane, (e) Arg-phenethylamide, HBTU, HOBt, DIPEA, DMF, (f) TFA, precipitation in diethyl ether, (g) Boc-Arg-OH, HBTU, HOBt, DIPEA, DMF, (h) TFA, precipitation in diethyl ether, purification by HPLC.

### Preparation of Peptide **109**

A laboratory-scale process for the preparation of peptide **109** is outlined in Scheme 2. All amino acids used in peptide **109** were enantiomerically pure with an l-configuration.

At this point, it may be pertinent to quickly summarize the simplification that has been achieved in terms of size and antimicrobial efficacy through the drug development process. In Table 6, important molecular properties and the antibacterial efficacy for lactoferricin B, LFB and peptide **109** are compared.

**Table 6:** Molecular Mass, Number of Residues and MIC Values in mg/L or (μM) against *S*. *aureus* and *E. coli* of Lactoferricin B, LFB, and Peptide **109** ^a^

peptide	lactoferricin B^17^	LFB^9^	**109**
Molecular mass	3126	2065	788
Number of residues	25	15	3
MIC *S. aureus*	10 (30)	100 (48)	4 (5)
MIC *E. coli*	10 (30)	50 (24)	8 (10)

a *S. aureus* ATCC 25923 and *E. coli* ATCC 25922.

Table 6 reveals that the MIC values when measured by mass (mg/L) appear not to have been improved very much (from 10 to 8 and 4) by going from lactoferricin B to peptide **109**; the significant reduction in molecular size makes peptide **109** 3–6 times more active than lactoferricin B (depending on the bacteria tested) when MIC is measured by concentration (μM). The MIC value (measured in mass units) is almost invariant between lactoferricin B and peptide **109** despite the large simplification of the molecular structure. This observation is interpreted as an indication that the MOA for AMPs as well as SAMPs is not dependent on a specific target but rather a consequence of the peptides instead attacking a less specific target covering the microbe.

The conclusion that can be drawn from Table 6 is that peptide **109**, with a 4 times reduction in mass and an 8 times reduction in number of residues, is a molecule with much improved antibacterial efficacy compared to lactoferricin B. However, the total mass of peptide required to kill the bacterium is only slightly lower.

### Antimicrobial Spectrum of Peptide **109**

One of the first tasks in the documentation of the lead candidate was to investigate the antimicrobial spectrum of **109** in more detail. First, a broad set of microorganisms was tested for susceptibility by determining the MIC value against **109**. The microorganisms in this library were all clinically relevant and encompassed both bacteria (Gram-positive and negative) and fungi (yeasts and filamentous fungi). The results from the broad screening are summarized in Table 7 and show broad activity against Gram-positive, Gram-negative, and fungi at low MIC values. However, some Gram-negative bacteria (e.g., *Proteus mirabilis*) had a much wider range of MIC values.

**Table 7:** Antimicrobial Activity against a Panel of Microbes Measured as MIC in mg/L for peptide **109**, Levofloxacin against Bacteria, and Amphotericin B against Fungi

microorganism	comment	**109**	comparator
Gram-Positive Bacteria			Levofloxacin
*S. aureus*	Antibiotic susceptible	2–4	0.12–0.25
	Resistant (MRSA, VISA, SSCmec)	4–8	0.12–8
Other *Staphylococci*	*S. epidermidis, S. hemolyticus*	1–8	0.12–0.5
*Enterococcus spp*	*E. faecialis, E. faecium* (antibiotic susceptible and resistant)	4–16	0.5–64
*S. pneumoniae*	Antibiotic susceptible and resistant	16–32	0.5–1
β-hemolytic *Streptococcus*	*S. pyogenes, S. agalacticae*, Group C and Group G clinical isolates susceptible and resistant	8–32	0.25–1
Other Gram-positive	*C. jeiceium, L. monocytogenes, P. acnes*	8–32	0.25
Gram Negative			Levofloxacin
*E. coli*	Antibiotic susceptible and resistant	8	≤0.06–8
*K. aerogenes*	Antibiotic susceptible and resistant	16	≤0.06–0.25
*Enterobacter* sp	Antibiotic susceptible and resistant	4–16	≤0.06
*Salmonella* sp	Antibiotic susceptible and resistant	8	≤0.06
*P. mirabilis*	Antibiotic susceptible and resistant	16 → 128	≤0.06
*P. aeruginosa*	Antibiotic susceptible and resistant	4–8	1–2
Other Gram-negative		8 → 128	≤0.06–4
Anaerobes			Levofloxacin
*C. difficile*	Antibiotic susceptible	16	4
Fungi			Amphotericin B
*C. albicans*		8	0.5
*A. niger*		8	0.5
*T. interdigitale*		8	0.5

This initial screening was followed by a second screening against a series of clinical isolates (30 or 50) of each strain selected between Gram-positive and Gram-negative bacteria: *S. aureus*, MRSA, *S. epidermidis*, MRSE, Group A, B, and C *Streptococci*, *E. faecalis*, and *E. faecium* (both *Enterococci* species were represented with vancomycin sensitive (VS) and resistant (VR) variants). The Gram-negative strains were represented with *P. aeruginosa*, *E. coli*, *K. pneumoniae*, and an *Enterobacter spp*. panel. The antimicrobial efficacy of peptide **109** was described by its MIC values. The result of this screening is shown in Figure 3.

**Figure 3:**
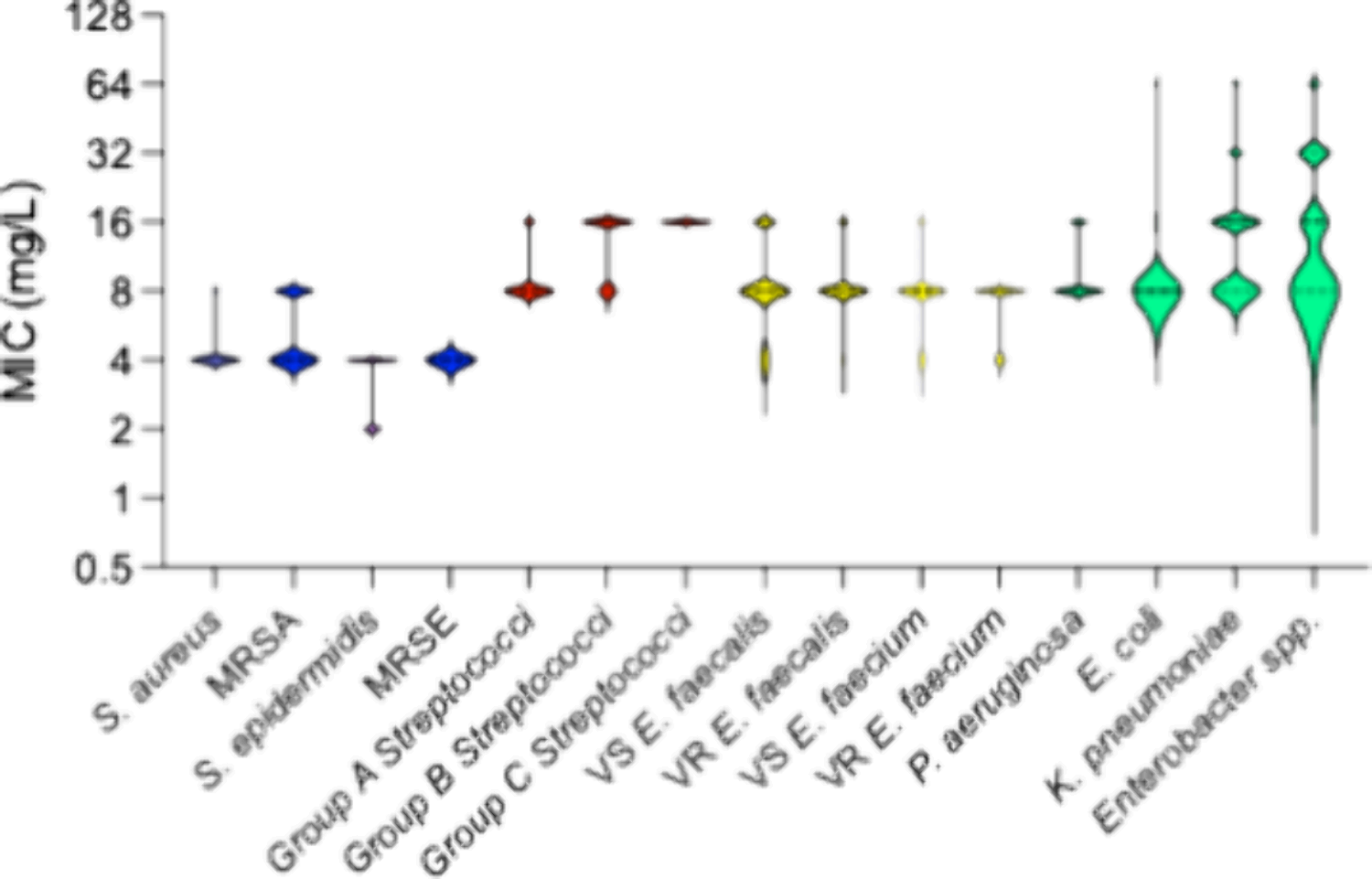
A violin plot of the screening of peptide **109** against a panel of clinical isolates (30–50) for each strain. The width of each violin lane corresponds to the portion of isolates with MIC values at that particular concentration.

The screening against clinical isolates revealed several interesting features. First, the activity spectrum of **109** is very broad, a finding that is consistent with naturally occurring AMPs. Second, the lane for each Gram-positive strain is very short and concentrated on one, or maximum two, MIC values, thus showing very little variation within the isolates. Furthermore, there is no difference between the drug-resistant strains and the strains susceptible to antibiotics, confirming that no cross-resistance was observed. Concerning the *Streptococci*, Group A is more sensitive than Group C, whereas Group B assumes an intermediate position. The Gram-negative bacteria reacted similarly as the Gram-positives to **109**, with the marked exception of the heterogeneous collection of *Enterobacter spp*. that showed a more prolonged lane with a few organisms at 4× and 8× the minimal MIC observed. All in all, the results from the screening of clinical isolates were very pleasing from a drug development point of view, as they corroborated that the truncation of lactoferricin B in the development process had not destroyed the broad range of antimicrobial activity belonging to the class of natural AMPs. The variety of strains susceptible to **109** and the very short MIC span experienced for each strain clearly show that the class of SAMP molecules behave in the same fashion as the natural AMPs.

To further substantiate that **109** showed no cross resistance against commonly used antibiotics, a screen of 50 clinical isolates of MRSA and 50 clinical isolates of MRSE against peptide **109** and 10 commonly used antibiotics spanning various antibiotic classes was performed.

The data in Table 8 show that the MIC values for peptide **109** changed very little (from 2 to 8 mg/L for MRSA, and constant 4 mg/L for MRSE) when moving from the most sensitive (**MIN**) to the least sensitive isolate (**MAX**) of each species. For all other antibiotics except vancomycin, full resistance against erythromycin, levofloxacin, and cefotaxime is found for 50% of the isolates. At least 10% of the isolates were resistant to all antibiotics tested other than **109** (data not shown). These data strongly suggest that **109** does not have cross resistance to any of the antibiotics screened, and that **109** has similar activity toward the clinical isolates irrespective of their resistance pattern. Further studies of the activity of **109** against even more resistant Gram-positive clinical isolates has been published elesewhere.^18^

**Table 8:** Antimicrobial Efficacy Measured as MIC in mg/L for 50 Clinical Isolates of MRSA and 50 Clinical Isolates of MRSE against Peptide **109** and 10 Different Commonly Used Antibiotics^a^

		MIC mg/L			
pathogen (N)	antimicrobial	MIN	50%	90%	MAX
Methicillin-resistant *Staphylococcus aureus* (MRSA) **(50)**	**109**	**2**	**4**	**8**	**8**
	Amoxicillin	8	≥32	≥32	≥32
	Clindamycin	0.06	0.12	≥16	≥16
	Erythromycin	0.25	32	32	32
	Gentamicin	≤0.06	0.25	1	≥64
	Imipenem	0.06	4	≥16	≥16
	Levofloxacin	0.12	16	≥32	≥32
	Mupirocin	≤0.06	0.12	1	≥32
	Cefotaxime	8	≥64	≥64	≥64
	Tetracycline	≤0.12	0.25	1	32
	Vancomycin	0.25	0.5	0.5	2
Methicillin-resistant *Staphylococcus epidermidis* (MRSE) (**50**)	**109**	**4**	**4**	**4**	**4**
	Amoxicillin	1	8	≥32	≥32
	Clindamycin	≤0.03	0.06	≥16	≥16
	Erythromycin	≤0.12	32	32	32
	Gentamicin	≤0.06	16	≥64	≥64
	Imipenem	≤0.03	0.25	≥16	≥16
	Levofloxacin	0.06	4	16	≥32
	Mupirocin	≤0.06	0.12	≥32	≥32
	Cefotaxime	0.25	4	≥64	≥64
	Tetracycline	≤0.12	1	2	32
	Vancomycin	1	1	2	2

a The MIC-columns describe the minimum MIC found, the MIC value killing 50% and 90% of the isolates, and the maximum MIC value recorded for each active molecule.

### Peptide **109** Kill Kinetics

One defining trait for natural cationic AMPs is their rapid membranolytic effect.^19^ It is thus important to establish that peptide **109** has the same rapid mode of action (MOA) as the natural AMPs as a rapid killing rate, which is partly responsible for the lack of resistance development in the microorganisms. The kill kinetics of **109** at 8, 2, and 0.5 times the MIC were tested against the *S. aureus* FDA486 strain with oxacillin and vancomycin as positive controls. The kinetic effect of **109** and the controls is shown in Figure 4. An increasingly rapid lytic effect of **109** against *S. aureus* at 2 and 8 times MIC ending in a six-log reduction in CFU is in line with natural AMPs, whereas sub-MIC levels of **109** and 8 times MIC of the control antibiotics only reduce the CFU with one log in 5 h. It is also interesting to note that a similar fast killing against the human yeast pathogen *C. albicans* has also been observed.^20^

**Figure 4:**
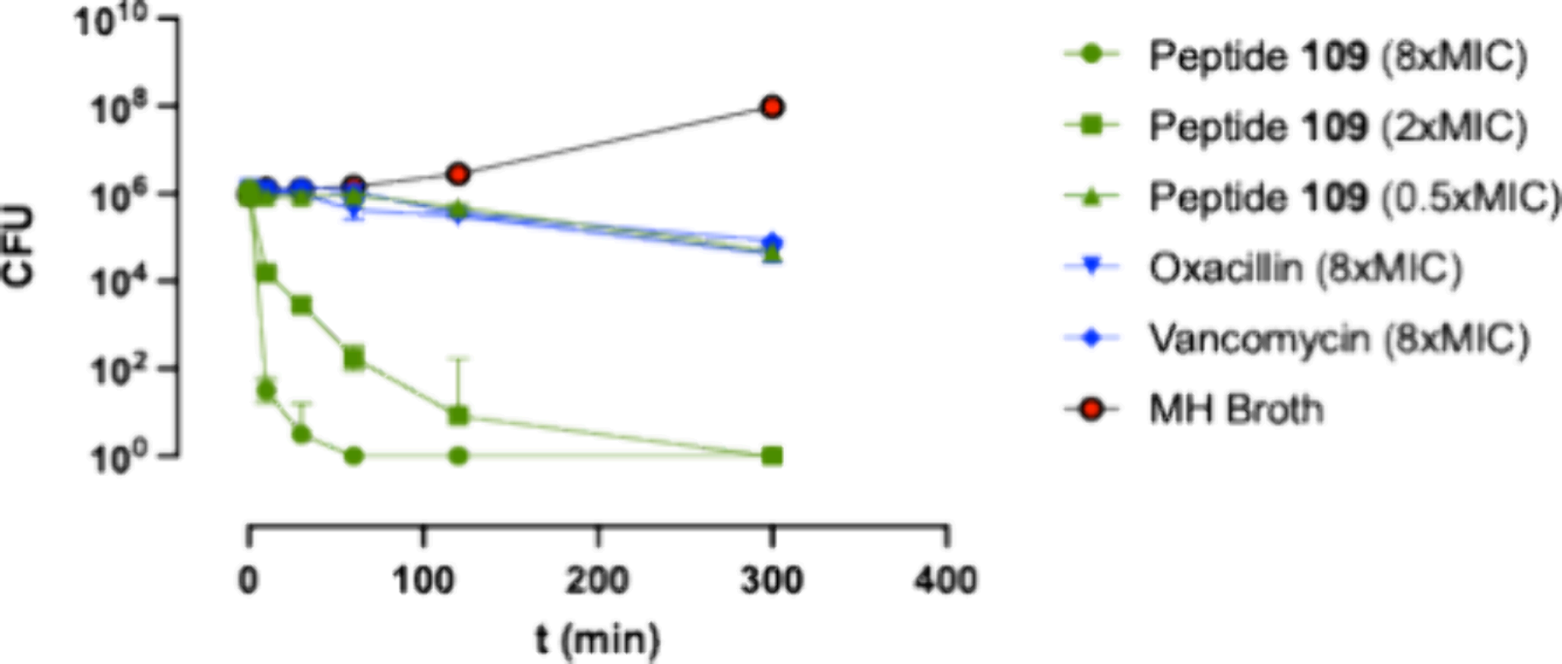
Kill kinetics measured as colony forming units (CFU) vs time of peptide **109**, oxacillin, and vancomycin on *S. aureus* FDA486.

### Peptide **109** Efficacy against Biofilms

In the majority of infections in humans, the bacteria form biofilms. The National Institutes of Health (NIH) estimate that in microbial infections 65% are associated with biofilm formation, and in chronic infections the number approaches 80%.^21^ Bacterial biofilms are aggregated communities of bacteria that live in a self-produced matrix and represents severe challenges for clinically used antibiotics.^22^ In a biofilm, most bacteria develop a tolerance to the antibiotics,^23^ a trait that can make treatment of biofilm a challenge. Antimicrobial peptides poses an intrinsic activity against biofilms, and AMPs have been proposed as an alternative to common antibiotics in the treatment of biofilm infections.^24^ Peptide **109** has been evaluated in an *in vitro Staphylococcus* biofilm model,^25^ and it showed that the peptide could completely eliminate metabolic activity in the biofilms at concentrations <10 times the planktonic MIC, whereas classical antibiotics did not eliminate metabolic activity even at concentrations >100 times the planktonic MIC. The encouraging results on Gram-positive bacteria were followed up in *P. aeruginosa*, a Gram-negative pathogen. The model used in the *P. aeruginosa* experiment was a flow chamber allowing for fresh nutrients, as described in the literature.^26^ Viable *P. aeruginosa* bacteria were visualized with GFP tagging or Syto9 and dead cells stained with propidium iodide; hence, living biofilm will appear green and dead biofilm red. The *P. aeruginosa* strain used (SM2467) had a planktonic MIC of 14 mg/L, and mixing **109** at this concentration distinctly affected the biofilm (Figure 5B compared with Figure 5A), but the biofilm was clearly composed of living cells. At 2-fold the planktonic MIC, the bottom of the biofilm was clearly dead (Figure 5C). At 4-fold MIC the biofilm was dead and started to disintegrate (data not shown). Thus, peptide **109** functions similarly to natural AMPs with regard to its efficacy against biofilms.

**Figure 5:**
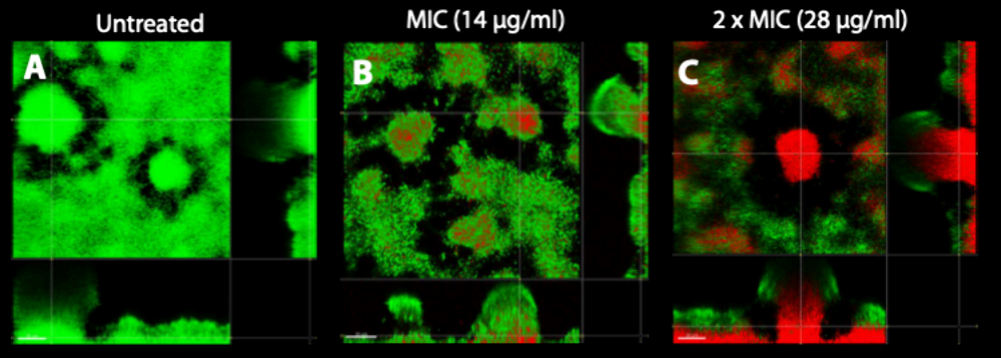
Confocal laser microscopy imaging in the flow cell biofilm system with *P. aeruginosa* SM2467 as a test organism. Panel A shows an untreated biofilm, and panels B and C show the effect of 14 and 28 mg/L of peptide **109**, respectively, equivalent to 1 and 2 times the planktonic MIC.

### Efficacy of Peptide **109** under High Salt Situations

A common challenge for natural AMPs is the loss of antimicrobial activity in the presence of typical host factors such as conditions with high salt concentrations.^27,28^ This effect may in particular be a challenge in the presence of divalent cations such as Mg^2+^ and Ca^2+^. An experiment where **109** was tested against *S. aureus*, *S. epidermides*, *E. coli*, and *P. aeruginosa* in the presence of an increasing amount of sodium ions as well as a 2:1 calcium/magnesium combination at a concentration mimicking blood plasma showed an almost total resilience of the antibacterial activity in the presence of a high concentration of sodium ions, where a 2-fold loss of efficacy was observed (Table 9). As this loss of activity is within the error typically found in these experiments, any loss of activity due to added ions is debatable. The effect of the addition of divalent cations corresponding to those found in blood was also very small.

**Table 9:** Antimicrobial Efficacy Measured as MIC in mg/L for a Series of Bacteria As a Function of External Salt

	NaCl concentration				divalent cation salt
bacterium	0 mM	10 mM	50 mM	150 mM	2 mM CaCl_2_ + 1 mM MgCl_2_
*S. aureus* *NCTC 8325*	1	2	2	2	2
*S. epidermidis* *ATCC 35984*	1	1	1	1	1
*E. coli* *ATCC 25922*	2	2	2	4	4
*P. aeruginosa* *CCUG 49694*	4	4	8	8	8

### Peptide **109** – No Propensity for Resistance Development Found

An important property of natural AMPs is their resilience toward resistance development. As mentioned in the Introduction, it is very likely that the presence of AMPs spans eons, and as a class of molecules, the AMPs are probably evolutionarily older than 500 million years. It is thus probable that even if AMP molecules themselves have evolved over the eons, it is also likely that their basic MOA, lysing the microbial membrane, has been retained. The AMPs of today are thus living proof that the MOA of these molecules provides the peptides with a killing ability that is extremely difficult for microorganisms to develop resistance against. Peptide **109** is designed from important characteristics discovered in, and derived from, the natural AMP. Furthermore, as **109** shares many properties with the natural AMPs efficacy-wise, it is tempting to believe that **109** shares the MOA with the natural AMPs, and hence peptide **109** will also show a similar resilience to resistance development. The inherent problem with experiments involving resistance development is that the negative, the absence of resistance development, cannot be proved; only the positive, the existence of resistance development, can be established experimentally. In addition, the study of resistance development is further complicated by the sheer number of methods available. The rate of spontaneous resistance (mutation frequency) can be estimated by finding the number of inherently resistant (actually less susceptible) microbes in a large pool (10^9^–10^10^) of organisms. Another method involves trying to force resistance development in the bacteria through multiple passes over an increasing concentration of **109**.

The spontaneous resistance rate was determined as the frequency of bacterial colonies showing resistance, as evidenced by finding colonies able to grow on agar plates containing various multiples of the MIC (data compiled in Table 10). The selection spontaneous resistant bacteria were made on plates containing 2, 4, and 8 times the determined MIC for a particular strain by applying concentrated suspensions of bacteria at approximately 10^10^ per plate. If confluent growth occurred in any of the agar plates, a random selection was taken from the plate and used to reinoculate agar containing an antimicrobial at the same concentration as the original selection. Resistance was confirmed if growth occurred also after subculturing and incubation. Fusidic acid was used as a positive control and showed confluent growth, i.e., resistance, in all experiments. Peptide **109** did show one instance of confluent growth (4× MIC *S. aureus* VRS1), but the finding could not be confirmed as resistance by subculturing and was rather labeled as tolerance instead of spontaneous resistance. In conclusion, no spontaneous resistance for *S. aureus* strains against **109** could be found, irrespective of the resistance pattern of the strains used. The lack of finding spontaneous resistance in drug resistant strains is a clear sign of a lack of cross resistance between peptide **109** and methicillin, vancomycin, and teicoplanin.

**Table 10:** Mutation Frequencies in *S. aureus* Estimated after Selection on Agar Plates Containing Peptide **109** at 2×, 4×, and 8× MIC

*S. aureus* strain	resistance pattern	2× MIC	4× MIC	8× MIC
ATCC 29213	Susceptible reference strain	<1.5 × 10^–9^	<1.5 × 10^–9^	<1.5 × 10^–9^
ATCC 43300	Methicillin-resistant reference strain	<1.5 × 10^–9^	<1.5 × 10^–9^	<1.5 × 10^–9^
Mu50	Vancomycin-intermediate resistant strain	<2.8 × 10^–9^	<2.8 × 10^–9^	<2.8 × 10^–9^
VRS1	Fully vancomycin resistant strain	<2.7 × 10^–9^	CF^a^	<2.7 × 10^–9^
GP06	Teicoplanin-intermediate resistant strain	<1.2 × 10^–9^	<1.2 × 10^–9^	<1.2 × 10^–9^

a Confluent growth obtained, but MIC could not be confirmed by subculture at concentration of initial selection.

The forced resistance experiment was performed for 14 passes using 0.5× MIC at each pass; i.e., the MIC was determined for each pass, and the culture was treated with 0.5× this MIC value. The same susceptible and drug-resistant strains of *S. aureus* strains as above was used in the forced resistance test and fusidic acid used as control. The experiments showed no resistance development for any of the strains, neither wild type nor drug resistant, against **109**. A typical result from the forced resistance experiment using **109** and fusidic acid, in this example against the Mu50-strain, is shown in Figure 6. Fusidic acid, in contrast, readily developed resistance in all of the strains tested.

**Figure 6:**
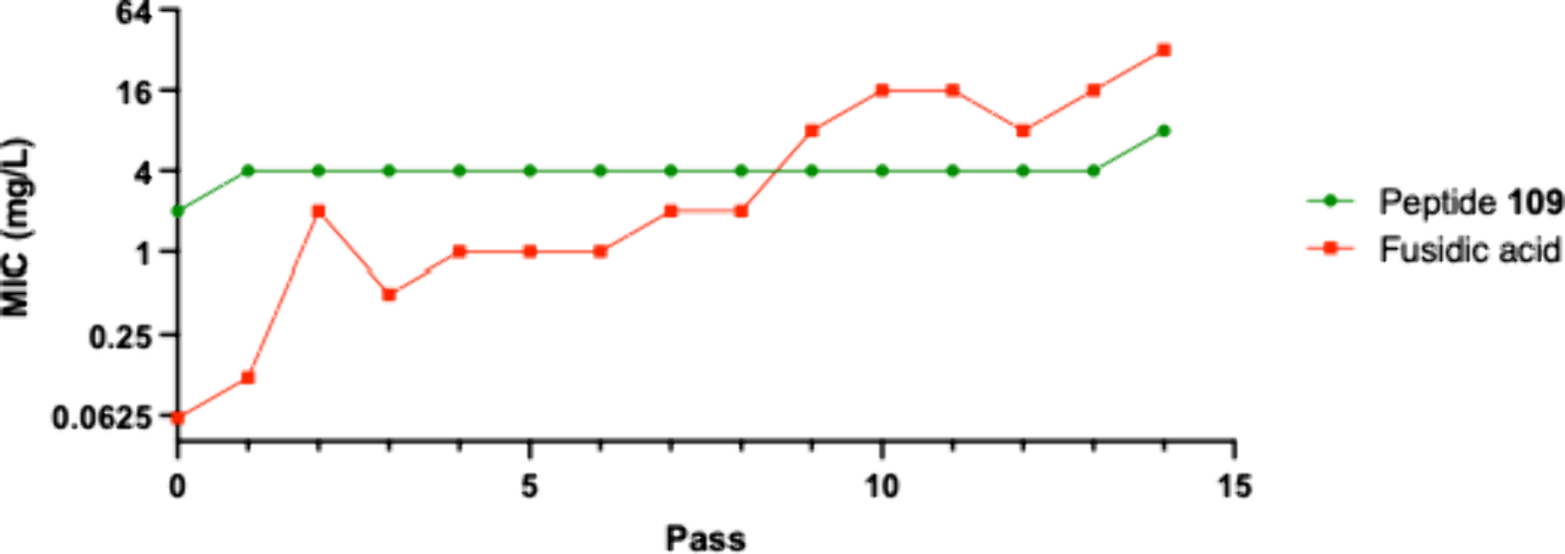
MIC versus pass in a forced resistance experiment toward peptide **109** and fusidic acidic on the *S. aureus* Mu50 strain.

As previously stated, it can never be concluded that resistance to an agent will not occur clinically. However, the data above show that antimicrobial peptide **109** is not predisposed to resistance development by the methods employed. Other groups have examined the resistance development using similar methods for even longer periods (26 passes, *E. coli*, *S. aureus*, and MRSA,^29^ 60 passes, *S. aureus* ^30^) also without detecting any resistance development.

### Antiviral Effects of Peptide **109**

It has been well established that some natural AMPs have good efficacy against encapsulated viruses.^31^ The target for the peptides is likely the membrane coating some viruses receive when budding out of an infected cell.^32^ These membranes have lost their asymmetric integrity typical for eucaryotes; hence, the encapsulated viruses are attacked at concentrations where eukaryotic cytotoxicity is not observed. In addition to this proposed MOA, other targets as well as immunomodulatory effects^33^ may also exist. *In vitro* studies revealed the convincing efficacy of **109** against influenza A/B, RSV, and SARS-CoV-2. The findings from the in vitro experiments were confirmed in ferret and hamster models for influenza A and SARS-CoV-2, demonstrating dose-dependent efficacy of peptide **109** in inactivating virus down to a concentration consistent with the *in vitro* data.

### Mode of Action against Bacteria

The interaction of natural AMPs with biological membranes depends on the lipids contained in the cell membrane itself,^34^ where individual AMPs interact with the bacterial cell membrane, thus interfering with the construction of the inner or outer bacterial membrane, resulting in cell death.^35^ The important matter is that the natural AMPs have a variable but certain selectivity for microbial membranes over our eukaryotic counterparts. As **109** has been developed based on principles derived from the natural AMPs but is considerably modified and shortened, it may be questioned whether the selectivity toward microbial membranes of the natural AMPs has been maintained. The MOA of **109** against *S. aureus* has recently been studied in great detail,^36^ and only highlights will be covered here. The *Staphylococcal* bacterial cell membrane has a substantial negative charge as shown by lipidomic studies,^37^ whereas the outer leaflet of the eukaryotic membranes is regarded as neutral. Molecular dynamics (MD) simulations using a coarse grain force field suggest that peptide **109** in aqueous media spontaneously aggregates into nanometer-sized clusters, where the hydrophilic positively charged side chains in the Arg residues are orientated toward the exterior and the bulky and lipophilic Tbt side chain resides on the inside of the clusters. The presence of these clusters has been observed experimentally using atomic force microscopy. The clusters have a highly cationic surface; thus, they interact electrostatically with the bacterial membrane, fusing with the membrane and inserting a cluster of **109** peptides. The peptide insertion at a high local concentration destabilizes the bacterial membrane leading to bacterial lysis. According to this MOA, **109** acts on the bacteria as a disinfectant, causing rapid lysis. But in contrast to disinfectants in general, **109** works with high selectivity against bacteria. This MOA coincides with many of the measured properties of the peptide, like rate of killing and a high propensity to withstand bacterial resistance development. In addition, it is found that peptide **109** has a profound effect on lateral lipid domains (membrane rafts), causing these to fuse and dissolve. As these domains are involved in β-lactamase derived resistance, synergistic effects between peptide **109** and β-lactam antibiotics in resistant *S. aureus* strains have been observed providing the potential to resensitize resistant strains to conventional antibiotics by the application of sub-MIC amounts of peptide **109**.

The MOA of peptide **109** thus represents a unique combination of apparently contradictory characteristics. Peptide **109** appears to have Janus-faced properties,^500^ behaving like a disinfectant with effective killing of microorganisms without provoking antimicrobial resistance combined with a profound selectivity against microbes over human cells.

### *In Vivo* and *Ex Vivo* Studies of Peptide **109**

Originally, peptide **109** was targeted as a topical drug against skin infections. The peptide **109** is water-soluble and is easily formulated as a hydrogel. The hydrogel formulation has been tested in both *ex vivo* and *in vivo* models. The results from the *ex vivo* models against fungi (*C. albicans* and *Trichophyton rubrus*) have been published^38^ and reveal excellent activity compared to commercial drugs in a candidiasis skin model and in a nail fungus model. The formulated peptide has also been studied in a variety of bacterial skin infection models, of which some can be found in the literature.^39,40^ The findings are generally in line with the expectations. A gel formulation of peptide **109** is fast acting and effective, much faster than classical antibiotics. A typical example from a bacterial skin infection model is shown in Figure 7.

**Figure 7:**
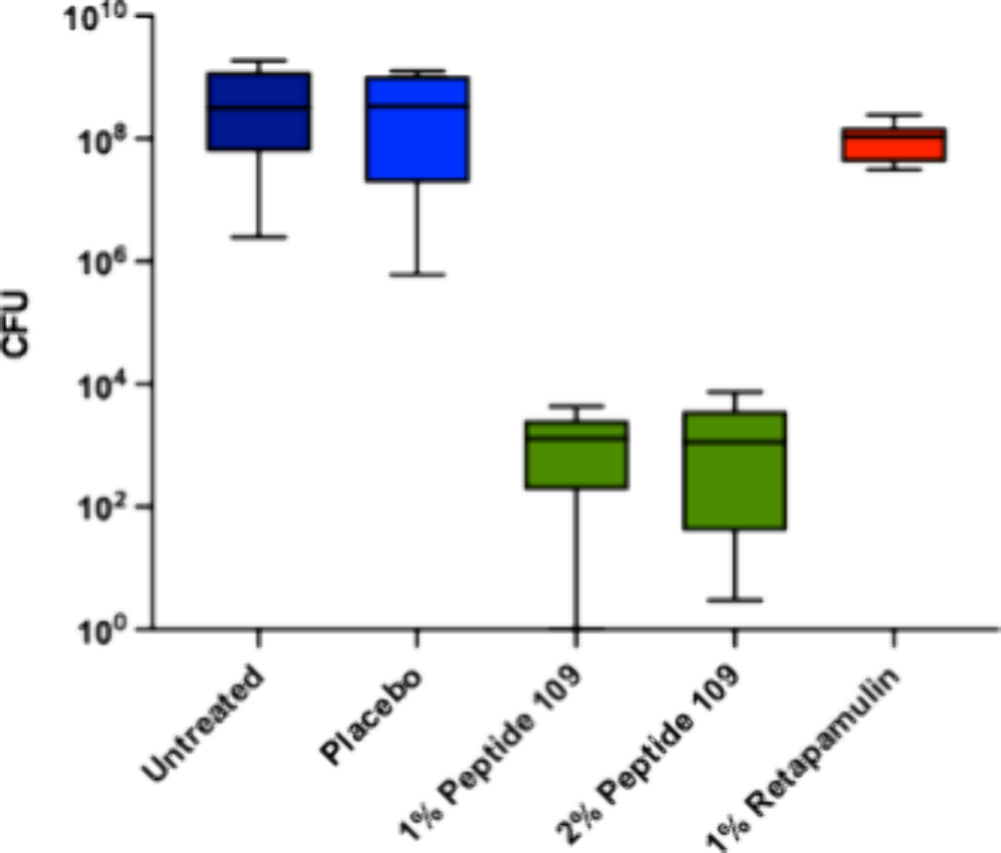
TID (*ter in die*, three times a day) single day dosing of 1% and 2% peptide **109** in a hydrogel formulation and 1% retapamulin in a *S. aureus* infection model in mice.

The results from this study clearly reveal the effectiveness of peptide **109** in the infection model, with 2% of **109** being marginally better than the 1% treatment. In contrast, 1% retapamulin treatment is inefficient in clearing the infection during the timespan of the experiment.

### Toward Clinical and Medical Device Use

The peptide **109** has received the INN name voxvoganan, which is used henceforth in the article. The road toward commercialization of voxvoganan was started in 2003 by Lytix Biopharma focused on pharmaceutical applications. The molecule has since split in two directions: as an API for use in medical devices by Amicoat since 2014, and for pharmaceutical applications by Pharma Holdings since 2017. Despite the division into two different fields of interest, the commercialization avenues for both companies share much of the preclinical work as the concerns for safety and toxicity are similar among them. Pharma Holdings was at the outset focused on a topical hydrogel formulation of voxvoganan, designed for the decolonization of resistant bacterial strains such as *S. aureus*, *Strep. pneumoniae*, *H. influenzae*, and *P. aeruginosa*, but has since shifted focus toward the antiviral properties of voxvoganan. Amicoat is developing voxvoganan as an API for integration as an antifouling agent against microbial colonization and biofilm formation in medical devices.

### Preclinical Studies Elucidating Toxicology and Safety

Originally, voxvoganan was developed as a drug for topical treatment of skin and surface infections, and comprehensive preclinical studies were performed. An excerpt of these is referred to in the next section and in Table 11. The ADME characteristics of voxvoganan was investigated *in vivo* with both IV and SC administration in mice, along with several *in vitro* tests on various cell lines and tissue samples.

**Table 11:** Partial Overview of Preclinical Studies Performed on Voxvoganan

title	study type	model	dose range/route
Efficacy of LTX-9 against *Staphylococcus aureus* E2371 and *Staphylococcus aureus* FDA486 in the murine peritonitis model	Pharmacology - Primary Pharmacodynamics	Mice	30 mg/kg - SC
Determination of the effects of an antimicrobial peptide on *S. aureus* proliferation and wound healing in a porcine deep partial thickness wound model (pilot study)	Pharmacology - Primary Pharmacodynamics	Pigs	1, 2, and 5% - dermal
Determination of MIC, bactericidal activity and resistance development with Ltx-109	Pharmacology - Primary Pharmacodynamics	Panel of clinical isolates	N/A
Effect of Ltx-9 on the Modified Irwin Screen Test in the Rat	Pharmacology - Safety Pharmacology	Rats	0, 0.75, 3.75, and 7.5 mg/kg - IV
LTX-109: Evaluation by Intravenous Administration on Blood Pressure, Heart Rate and Lead II ECG and Respiratory Function in Conscious Telemetered Beagle Dogs	Pharmacology - Safety Pharmacology	Dogs	1, 2.5, 5 mg/kg - IV
Effect of LTX-109 on hERG Tail Currents Recorded from Stably Transfected CHO Cells	Pharmacology - Safety Pharmacology	CHO cells stably transfected with hERG	N/A
Pharmacokinetics of Ltx-9 in mice following single dose administration of 15 mg/kg intravenous and 30 mg/kg subcutaneous	Pharmacokinetics - Absorption/kinetics parameters	Mice	15 mg/kg – IV30 mg/kg – SC
Exploratory subcutaneous pharmacokinetic study of Ltx-5; Ltx-7 & Ltx-9 at 20, 40, 40 mg/kg in mice, respectively	Pharmacokinetics - Absorption/kinetics parameters	Mice	40 mg/kg – SC
Quantitative whole body autoradiography study in mice after single intravenous administration of 3H-labeled Ltx 9.	Pharmacokinetics - Distribution	Mice	5 mg/kg - IV
Determination of binding of LTX-109 to cells in human blood	Pharmacokinetics - Distribution	Human blood	N/A
Determination of the *In Vitro* Binding of [^14^C]-LTX109 to the Plasma Proteins and Blood Cells in Rat, Dog and Human	Pharmacokinetics - Distribution	Rat, dog, and human blood	N/A
Evaluation of the Potential Induction Effect of LTX-109 on Cytochrome P450 CYP1A2, CYP2B6 and CYP3A4/5 Enzyme Activities in Freshly Isolated Human Hepatocytes	Pharmacokinetics - Metabolism	Human hepatocytes	N/A
Investigation of the Potential Inhibitory Effect of LTX-109 on Human Cytochrome P450 (CYP) Model Substrates	Pharmacokinetics - Metabolism	Human liver microsomes	N/A
Tissue, Species and Gender Variation in the In vitro Metabolism of [^14^C]-LTX-109	Pharmacokinetics - Metabolism	Rat, pig, and human	N/A
Acute toxicity of LTX-5, LTX-7, and LTX-9 following intravenous and subcutaneous administration in mice	Toxicology - Single-Dose Toxicity	Mice	40 mg/kg – SC20 mg/kg - IV
LTX-9:14 Day Repeat Study by Dermal Administration in Rats with a 14 Day Recovery Period	Toxicology - Repeat-Dose Toxicity	Rats	0, 1, 2, and 5% - dermal
LTX-109: Single and 7 Day Repeat Intravenous Dose Range Finding Study in Rats	Toxicology - Repeat-Dose Toxicity	Rats	2.5, 5, 7.5, and 10 mg/kg – IV Single-dose0.5, 2.5, and 5 mg/kg – IV Repeat-dose
LTX-109: Single and 7 Day Repeat Dermal Dose Range Finding Study in Rats	Toxicology - Repeat-Dose Toxicity	Rats	0, 1, 2, and 5% - dermal
LTX-109: Intravenous Toxicokinetic Study in Dogs	Toxicology - Repeat-Dose Toxicity	Dogs	0.5, 1, 2.5, and 5 mg/kg - IV
Ltx-9: Single and 7 Day Repeat Dermal Dose Range Finding Study in Mini-pigs	Toxicology - Repeat-Dose Toxicity	Pigs	0, 1, 2, and 5% concentration - derma
Ltx-9:14 Day Repeat Dermal Study in Mini-pigs with a 14 Day Recovery Period	Toxicology - Repeat-Dose Toxicity	Pigs	0, 1, 2, and 5% - dermal
LTX-5, LTX-7, and LTX-9: *In Vitro* Mutation Screening Test using Mouse Lymphoma L5178Y Cells.	Toxicology - Genotoxicity	L5178Y Mouse lymphoma cells	N/A
LTX 9-11R: Testing for Mutagenic Activity with *Salmonella typhimurium* TA 1535, TA 100, TA 1537, TA 98, and TA 102 (Antibiotic)	Toxicology - Genotoxicity	*Salmonella typhimurium*	N/A
LTX 9: Chromosomal Aberrations Assay with Chinese Hamster Ovary Cell Cultures In Vitro	Toxicology - Genotoxicity	CHO 10 B_4_	N/A
LTX 9: Mouse Lymphoma Mutation Study	Toxicology - Genotoxicity	Lymphoma cells	N/A
LTX 9: Micronucleus Test in Bone Marrow Cells of CD Rats: Bolus Intravenous Dosing and 24 and 48 h Sampling with Toxicokinetic Blood Sampling	Toxicology - Genotoxicity	Rats	2.5, 5, and 7.5 mg/kg - IV
LTX-109: A 14-Day Toxicity Study with Recovery in 8-Week Old Juvenile Minipigs	Toxicology - Reproductive and Developmental	Pigs	0, 1, 2, and 5% - dermal
LTX-109: Local Lymph Node Assay in the Mouse	Toxicology - Local Tolerance	Mice	0, 2, and 5% - dermal
Evaluation of the Potential for LTX-315 and LTX-109 to Induce Extravasation of Evan’s Blue Dye in Guinea Pig Skin	Toxicology - Other Toxicity Studies	Guinea pigs	0.2, 1, and 2 mg - intradermal
Evaluation of the Potential for LTX-315 and LTX-109 to Induce Passive Cutaneous Anaphylaxis in the Guinea Pig	Toxicology - Other Toxicity Studies	Guinea pigs	10 μg - intradermal

The absorption and pharmacokinetic profile were investigated by administering voxvoganan IV or SC to mice, and the concentration of voxvoganan in plasma and urine was examined at various time points up to 180 min post administration. Voxvoganan showed a short elimination half-life (Table 5, IV: 71 min, SC: 156 min) and high plasma protein binding (90% protein binding). The high protein binding indicated that the main distribution compartment was blood plasma.

The absorption kinetics was followed up by an SC administration of a higher dose in mice, where the plasma concentration of voxvoganan was monitored up to 24 h post administration. The dose normalized area under the curve (AUC) was 1.32 and with a clearance of 0.76 L/h/kg. The distribution of voxvoganan was investigated *in vivo* (IV in mice) and *in vitro* to determine binding to plasma proteins and blood cells. The *in vitro* studies showed a very high binding to plasma (cf. above) and low to no binding to blood cells. In the *in vivo* study, the distribution of radioactively labeled voxvoganan was monitored for 6 days post administration, revealing that voxvoganan was rapidly cleared from circulation by both urinary and gastrointestinal tracts indicating that both were routes of elimination.

No indication of CYP inhibition or induction was found when the effect on human CYP model substrates of voxvoganan was investigated. Furthermore, minimal metabolism was observed in hepatocytes, skin discs, and whole blood from rats, minipigs, and humans.

As the MOA of voxvoganan is to disrupt the cell membrane of procaryotes, it is imperative to show that this action is not carried over to eukaryote cells, leading to toxicity in patients. The toxicology of voxvoganan was hence thoroughly investigated in a range of single-dose and repeat-dose preclinical studies. These studies include genotoxicity, single-dose toxicity, repeat-dose toxicity, local tolerance, and developmental toxicity encompassing both *in vitro* and *in vivo* studies in many different models and routes of administration.

Safety pharmacology was assessed using both *in vitro* and *in vivo* tests. *In vivo* Irwin tests in rats and dogs, and potential hERG effects *in vitro* were examined by studying the effect of voxvoganan on the hERG tail currents in stably transfected CHO cells. The hERG results showed a marginally higher blockade compared to vehicle alone at concentrations below 2000 ng/mL. In the modified Irwin screen test, dogs were administered voxvoganan in a single IV injection. The highest dose was associated with some behavioral changes, but no changes were observed at lower doses, and by 3 h post dose all dogs were free of any clinical signs.

Potential genotoxic effects were studied in several *in vitro* models and one *in vivo* study. An *in vitro* mutation screening test using mouse lymphoma L5178Y cells showed no increase in mutation frequencies. Voxvoganan induced neither any structural chromosomal aberrations nor any increases in polyploidy when tested against Chinese hamster ovary cells, and as a result, it was concluded to not be clastogenic. Potential genotoxicity was further investigated *in vivo* in a micronucleus test in bone the marrow cells of rats receiving intravenous administration of voxvoganan at 7.5 mg/kg. No micronuclei were induced after 24 and 48 h sampling post administration, leading to the conclusion that there was no genotoxic potential for voxvoganan.

The delayed contact hypersensitivity potential by topical application of a voxvoganan hydrogel in 2% and 5% concentrations was investigated *in vivo* by using a local lymph node assay in mice. As the stimulation index never exceeded 3 in any of the test subjects, voxvoganan was not considered to have potential for direct contact sensitization.

The maximum tolerated dose (MTD) for both native and hydrogel-formulated voxvoganan has been investigated. Beagle dogs were administered up to 5 mg/kg in a single escalating IV bolus dose with a 7 day wash-out period between each dose. No clinical signs of toxicity were observed below the highest dose. Hence, 2.5 mg/kg was considered the no-adverse effect level (NOAEL), while 5 mg/kg was considered MTD in this study.

Pigs receiving both a single 5% hydrogel dose and TID dosing for 7 days on both intact and abraded skin were not associated with any adverse dermal reaction or any signs of systemic toxicity. The MTD was considered 28 mg/animal, 1.87 mg/kg for a single dose, and 60 mg/animal/day at 4 mg/kg/day for repeat dosing.

The primary toxicity observed following dermal administration of voxvoganan has been mild and reversible application site reactions. Voxvoganan has shown limited skin penetration into the systemic circulation, consistent with the appearance of local rather than systemic effects.

The potential toxicity of a formulation developed for the treatment of respiratory diseases was assessed through intranasal administration of the voxvoganan formulation to rats TID for 14 days. The NOAEL was at least the maximum dose tested, 7.2 mg/kg/day, conferring a good safety record compared with the anticipated doses in clinical trials.

### Voxvoganan Clinical Studies

Voxvoganan is currently in development as a nasal spray for use against the influenza virus. Previously voxvoganan has been investigated in Phase I and IIa clinical trials in both healthy volunteers and patients with Gram-positive skin infections, impetigo, persistent MRSA/MSSA nasal carriage, hidradenitis suppurativa, and COVID-19. In total, 261 subjects have been exposed to voxvoganan, and the compound consistently demonstrated a favorable safety profile with negligible systemic absorption. Reported adverse events were generally mild, transient, and confined to the site of application, and no serious treatment-related adverse events were observed. An overview of the clinical studies is provided in Table 12.

**Table 12:** Overview of Completed Clinical Studies on Voxvoganan

study ID	phase	NTC/EudraCT No.	study title	subject exposure	study status
C08-109-001	I (FIH)	NA/2009-012381-31	A randomized, double-blind, placebo-controlled, ascending dose, Phase I study to evaluate the safety and tolerability of topical LTX-109 in healthy subjects.	Voxvoganan: 28 subjects; Placebo: 9 subjects	Completed
C10-109-02	I/IIa	NCT01158235/2010-019254-40	A randomized, double-blind, placebo-controlled, ascending dose Phase I/IIa study to evaluate the safety, tolerability and efficacy of topical LTX-109 in subjects nasally colonized with methicillin-resistant/- sensitive *Staphylococcus aureus* (MRSA/MSSA).	Voxvoganan: 18 subjects; Placebo: 6 subjects	Completed
C10-109-03	IIa	NCT01223222/2010-021438-68	A randomized, double-blind, placebo-controlled, Phase IIa pilot study to evaluate the safety, tolerability and efficacy of Lytixar (LTX-109) in patients with uncomplicated, gram-positive, skin infection.	Voxvoganan: 18 subjects; Placebo: 6 subjects	Completed
C12-109-04	II	NCT01803035/NA	A Phase II, Randomized, Double-blind, Placebo- controlled Study to Evaluate the Efficacy and Safety of Two Doses of LTX-109 (1% and 2%) Versus Placebo in Impetigo	Voxvoganan: 140 subjects; Placebo: 70 subjects	Completed
C20-109-06	I/IIa	NCT04767321/2020-003975-16	A Phase I/IIa, Randomized, Double-blind, Placebo- controlled Study to Evaluate the Safety and Exploratory Efficacy of 3% LTX-109 compared to Placebo for nasal decolonisation of *Staphylococcus* *aureus*	Voxvoganan: 11 subjects; Placebo: 4 subjects	Completed
C20-109-07	I	NCT04756336/2020-000042-34	Proof of concept study on LTX-109 for treatment of Hidradenitis suppurativa	Voxvoganan: 11 subjects	Completed
C22-109-08	IIa	2022-001938-11	A Phase IIa, Randomized, Double-blind, Placebo- controlled Study to Evaluate the Efficacy, Safety and Tolerability of 3% LTX-109 compared to Placebo for nasal decolonisation of *Staphylococcus aureus*	Voxvoganan: 19 subjects; Placebo: 9 subjects	Completed
C21-109-09/Pharma Holdings	IIa	NCT04854928/2021-000455-39	A double-blind, placebo-controlled, interventional parallel group study to evaluate the antiviral effect of a single nasal application of LTX-109 3% gel, in comparison to placebo gel, in subjects with COVID-19 infection	Voxvoganan: 12 subjects; Placebo: 11 subjects	Terminated early due to recruitment difficulties.

The first-in-human Phase I trial (C08-109-01, *n* = 32) confirmed a good local tolerability and negligible systemic absorption of topical voxvoganan gel (1–5%) on both intact and abraded skin.

In subsequent nasal decolonization studies, short-term eradication of MRSA/MSSA was observed with 2% and 5% concentrations. In C10-109-02 (*n* = 24), statistically significant reductions in nasal counts were demonstrated compared to placebo (*p* = 0.0014), with eradication achieved in most voxvoganan-treated subjects at Day 3. However, eradication was typically transient, with recolonization in most subjects after the initial response. Similar rapid, but largely nondurable decolonization was reported in intensive dosing studies C20-109-06 (*n* = 15) and C22-109-08 (*n* = 28). In the C20-109-06 study, subjects received four applications of 3% voxvoganan every 2 h. The highest eradication rate (73%, 8/11 subjects) was observed at 6 h, with 5/11 still culture-negative at 48 h and 4/11 subjects at Day 22. This regimen was safe and well tolerated, with only mild to moderate local reactions (swelling and pruritus) that resolved promptly. In C22-109-08 study, subjects with persistent MSSA carriage were treated with four initial doses of 3% LTX-109 within 4.5 h, followed by either two or four additional doses over 48 h. Voxvoganan produced a measurable decolonization effect from 4.5 h up to 7 days, although complete eradication during predefined windows was not achieved in most subjects. Safety remained favorable.

In skin infection studies, voxvoganan demonstrated a possible bacteriological signal. In C10-109-03 (*n* = 24) in patients with uncomplicated Gram-positive skin infections, outcomes were similar between groups, but numerically higher bacteriological responses were seen with voxvoganan. In the larger nonbullous impetigo study, C12-109-04 (*n* = 210), clinical and microbiological success rates favored 2% voxvoganan numerically over placebo, though the primary end point did not reach statistical significance (*p* = 0.0787). Subgroup analyses suggested a potential benefit in more severe disease. Safety was consistently acceptable, with no serious adverse reactions being reported. Exploratory studies in *hidradenitis suppurativa* (C20-109-07, *n* = 11) and *SARS-CoV-2* (C21-109-09, *n* = 23 evaluable) were limited by small sample sizes and did not demonstrate meaningful efficacy but again confirmed the product’s safety and tolerability.

In conclusion, voxvoganan has demonstrated a consistent safety and tolerability profile across clinical studies, with statistically significant antibacterial effects in nasal MRSA/MSSA decolonization and numerical improvements in skin infection studies, supporting continued clinical evaluation, including its ongoing development as an early treatment for respiratory viral infection in the multinational ECRAID-Prime Phase IIa study. Results from this study are expected in 2026.

### Development of Voxvoganan as an Antifouling API in Medical Devices

Healthcare associated infections (HAI) originating from medical devices colonized with microbes is a major problem on the healthcare system, in particular in intensive care units, where a major factor is the use of a medical device.^41^ There is, therefore, a need to develop a new generation of medical devices with an effective antifouling capacity. Such antifouling devices will inhibit microbial colonization on the surface, thereby precluding biofilm formation and significantly reducing the risk of patient infection and, finally, also lessening the incidence and burden of HAI. Technologies that can readily be adapted to a wide variety of medical devices are being developed, using voxvoganan as the active ingredient (API).

The antifouling application technologies developed rely on a slow leakage of voxvoganan to replenish the API lost from the material surface. Depending on the device material, environment, and desired antimicrobial lifetime, the replenishment of voxvoganan on the surface may be addressed with a variety of technologies. The application technologies can conveniently be sorted into three categories: coating, impregnation, and compounding. In the coating methods, the underlying material is coated with a thin layer of a voxvoganan-containing material. The coating material developed until now can be selected from silicone, polyester, or thermoplastic polyurethane. The coating technology is quite agnostic regarding the underlying material and can be the same (e.g., silicone on silicone), or it can be different (e.g., silicone on titanium). The coating is a powerful and flexible technology because it can be applied both to various materials and finished devices. As an illustrative example, TPU coatings formulated with voxvoganan have shown continuous release of the peptide for >50 days. Antimicrobial testing both as prepared and after 7 days of aging in PBS showed full elimination of bacteria with >7 log reduction in CFUs (Figure 8A–C).

**Figure 8:**
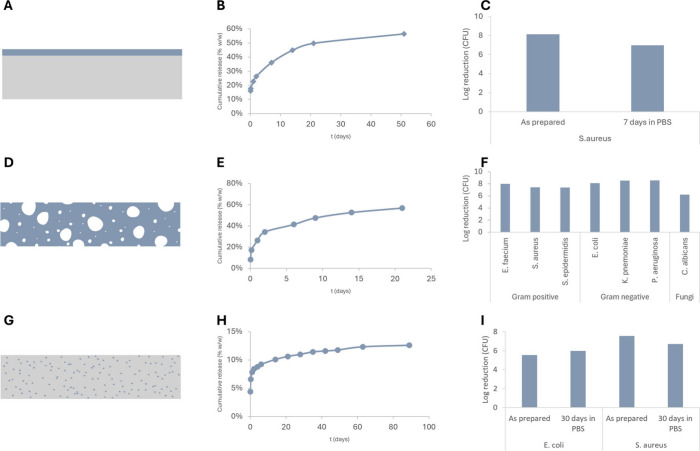
Illustration of different integration technologies coating, impregnation, and compounding with examples of corresponding release profiles and antimicrobial data. TPU coating (A), release profile in MQ-H_2_O (B) and log reduction as measured by a modified AATCC100 method on both fresh and aged samples (7 days in PBS) (C). Impregnated PU foam (D) with a corresponding release profile in PBS (E) and log reduction as tested by AATCC100 on a range of different microbes (F). Compounded and extruded silicone (G) with a corresponding release profile in PBS (H) and log reduction as measured by modified AATCC100 on both fresh and aged samples (30 days in PBS) (I).

The impregnation technologies are based on the ability of a solvent to reversibly penetrate a material. If the solvent contains voxvoganan, the solvent will drag the API into the medical device, as it infiltrates the material. When the device is removed from the solvent and the solvent has evaporated, the nonvolatile voxvoganan will remain in the device. The impregnation method can thus produce material with a gradient of voxvoganan, most on the outside and less on the inside, depending on the solvent penetration. The gradient obtained is dependent on the solvent, the concentration of voxvognan, and the duration of the impregnation process.

If the greater part of the API is at, or close to, the surface where the antifouling efficacy is desired, both coating and impregnation methods provide materials with a limited amount of voxvoganan, which is an asset from the point of view cost, but an inevitable limitation with regard to the lifetime of the treatment. As an example, a PU foam impregnated with voxvoganan showed continuous release for >20 days and a broad-spectrum activity in the standard wound care efficacy method AATCC-100 (Figure 8D–F).

In compounding or full material integration technologies where voxvoganan is combined with the raw materials used to produce the devices, the resulting raw material will have a homogeneous distribution of voxvoganan, and the medical devices produced using the full integration technology will have the antifouling API present irrespective of surface damage, cuts, and abrasions.

Two-component Pt-cured silicone (including LSR and HCR) can serve as an example of the compounding technology. Surprisingly, voxvoganan does not interfere with the Pt-curing catalyst, and it is thus feasible to mix the peptide into one of the LSR-ingredients prior to the formation of uncured silicone basis, device production, and curing. This method will result in a medical device, fully loaded with voxvoganan providing an antifouling efficacy lifetime in the range of months. HCR compounded with voxvoganan and extruded show a continuous release for >90 days and full antimicrobial activity both as prepared and after 30 days of aging in PBS (Figure 8G–I). An alternative technology based on the unusually high thermal stability of the API can also be used to create compounded materials. Biodegradable polyester materials like PLA, PGA, PDO, and combinations of these often used in absorbable medical devices can have melting points compatible with the voxvoganan peptide. Hence, combinations of voxvoganan and bioabsorbable polyesters can be extruded by using industrial methods.

As the antifouling efficacy is dependent on a continuous leakage of voxvoganan to the surface, the lifetime of the effect is dependent on both the amount of voxvoganan available to replenish the surface and the rate of liberation of voxvoganan from more interior regions to the surface. The coating and impregnation technologies will thus have their efficacy lifetimes limited by the amount of voxvoganan in the device, while the lifetimes of the compounding methods will be limited by the release rate. The antifouling properties of the compounded medical devices can thus be very long either by the material being degraded continuously liberating voxvoganan to exert its antifouling effect as with the bioabsorbable polyesters or dependent on the rate of leakage from the internal storage of voxvoganan to the surface, as in the case with silicone. A completely different avenue based on covalently attached voxvoganan has recently been explored as a long-lasting antifouling technology.^42–45^

## Conclusion

Voxvoganan is a first-in-class molecule, regarded as either a peptidomimetic drug or an API for use in medical devices. Voxvoganan is the outcome of focused research over several decades into translating the unique and advantageous properties of the natural AMPs into a typical small molecule framework, thus allowing for industrial production and enhanced stability toward degradation. Voxvoganan may be considered as a mimetic of the natural AMPs; however, the molecule is still a peptide, thus inherently degradable by hydrolysis to simple amino acids that, combined with the hydrophilic nature, discourages bioaccumulation. A robust and comprehensive series of preclinical studies have been conducted to evaluate the compound’s stability, microbiological activity, tolerability, and pharmacokinetics. These included standard characterization assays, *in vitro* MIC studies, cytotoxicity testing, and dermal irritation models. Notably, systemic uptake was shown to be negligible following topical application of the gel formulation, consistent with its nonsystemic profile. Importantly, voxvoganan displayed broad-spectrum bactericidal activity *in vitro*.

Furthermore, voxvoganan has been through formal safety and toxicology studies, has a filed drug master file, and has been through preclinical and clinical trials. The next step toward the market involves examination by regulatory authorities like FDA in a new drug application for the pharmaceutical use or as an API in combination device. During the next few years, products containing voxvoganan are expected to be available both as a nasal spray to avoid viral respiratory infections and as an integral part of a new class of medical devices with effective antifouling properties. Either way, voxvoganan may represent the first antimicrobial peptide that can reach patients to avoid or cure infections.

## Materials and Methods

### Chemicals

Protected amino acids Boc-Trp-OH, Boc-Arg-OH, and Boc-Arg-OMe were purchased from Bachem AG, while Boc-4-iodophenylalanine, was purchased from Aldrich. Isopropylamine, 2-phenylethylamine, and *n*-hexylamine used in modifying the C-terminus of the peptides, were purchased from Fluka. Diisopropylethylamine (DIPEA), 1-hydroxybenzotriazole (1-HOBt), and *O*-(benzotriazol-1-yl)-*N*,*N*,*N*′,*N*′-tetramethyluronium hexafluorophosphate (HBTU) were purchased from Fluka. 2-Naphthylboronic acid, tri-*o*-tolylphosphine, benzyl bromide, and palladium acetate were purchased from Aldrich. Solvents were purchased from Merck, Riedel-de Han, or Aldrich and used without further purification except for CH_2_Cl_2_, which was filtered through alumina before use.

### General Procedure for Suzuki–Miyaura Couplings

Benzyl Boc-4-iodophenylalanine (1 equiv), arylboronic acid (1.5 equiv), sodium carbonate (2 equiv), palladium acetate (0.05 equiv), and tri-*o*-tolylphosphine (0.1 equiv) were added to a degassed mixture of dimethoxyethane (6 mL/mmol of amino acid) and water (1 mL/mmol of amino acid). The reaction mixture was kept under argon and heated to 80 °C for 4–6 h. After being cooled to room temperature, the mixture was filtered through a short pad of silica gel and sodium carbonate. The filter cake was further washed with ethyl acetate and combined with the other fraction before the solvents were removed under reduced pressure. The products were purified using flash chromatography using mixtures of ethyl acetate and *n*-hexane as an eluent.

### General Procedure for De-esterification of Benzyl Esters

The benzyl ester was dissolved in DMF and hydrogenated for 2 days at ambient pressure and temperature using 10% Pd on carbon as catalyst. At the end of the reaction, the catalyst was removed by filtration, and the solvent was removed under reduced pressure. The free acid was isolated by recrystallization from diethyl ether.

### General Procedure for Solution Phase Peptide Synthesis Using HBTU as the Coupling Reagent

The peptides were prepared in solution by stepwise amino acid coupling using a Boc-protecting strategy according to the following general procedure. The C-terminal peptide part with a free amino group (1 equiv), Boc-protected amino acid (1.05 equiv), and 1-HOBt (1.8 equiv) were dissolved in DMF (2–4 mL/mmol of amino component) before addition of DIPEA (4.8 equiv). The mixture was cooled on ice before HBTU (1.2 equiv) was added, and the reaction mixture was agitated at ambient temperature for 1–2 h. The reaction mixture was diluted with ethyl acetate and washed with a citric acid solution (5%) (v/v), a saturated NaHCO_3_ solution, and brine. The solvent was removed under a vacuum, and the Boc-protecting group of the resulting peptide was deprotected in the dark using 95% TFA or acetyl chloride in anhydrous methanol.

### Peptide Purification and Analysis

The peptides were purified using reversed-phase HPLC on a Delta-Pak (Waters) C18 column (100 Å, 15 μm, 25 × 100 mm) with a mixture of water and acetonitrile (both containing 0.1% TFA) as eluent. The purity of the peptides was further analyzed by RP-HPLC using an analytical Delta-Pak (Waters) C18 column (100 Å, 5 μm, and 3.9 × 150 mm). All peptides were ≥95% by HPLC-analysis ().

### Preparation of Boc-l-Phe­[4-(2-naphthyl)]-OBn

The title compound was prepared in 68% yield from 2-naphthylboronic acid by using the general procedure for Suzuki–Miyaura couplings. Boc-Phe­[4-(2-naphthyl)]-OBn was isolated by the recrystallization of the crude product from *n*-heptane. Spectral data: ESMS 504.3 (calcd 504.2, M + Na^+^); ^1^H NMR (CDCl_3_) δ 1.36 (s, 9H), 3.08 (m, 2H), 4.61 (m, 1H), 4.98 (d, *J*) 7.8 Hz, 1H), 5.04–5.15 (AB system, *J*) 12.3 Hz, 2H), 7.08–7.95 (m, 16H); ^13^C NMR (CDCl_3_) δ 28.0, 38.0, 55.0, 67.0, 125.5, 125.7, 126.0, 126.3, 127.5, 127.7, 128.2, 128.4, 128.5, 128.6, 129.9, 132.6, 133.7, 135.1, 135.2, 138.1, 165.0, 172.0 ppm.

### Preparation of Boc-l-Phe­[4-(2-naphthyl)]-OH

The title compound was prepared in 68% yield from the benzyl ester using the general procedure for de-esterification. Spectral data: ESMS 414.2 (calcd 414.2, M + Na^+^); ^1^H NMR (CDCl_3_) δ 1.36 (s, 9H), 3.08–3.21 (m, 2H), 4.60 (m, 1H), 4.95 (d, *J*) 7.4 Hz, 1H), 7.19–7.94 (m, 11H); ^13^C NMR (CDCl_3_) δ 28.0, 38.0, 54.0, 125.5, 125.7, 126.0, 126.3, 127.6, 127.7, 128.2, 128.5, 130.0, 132.6, 133.7, 135.1, 138.0, 139.9, 155.5, 175.6 ppm.

### Preparation of Boc-L-2,5,7-tri-*tert*-butyl-tryptophan-OH

. The title compound was prepared in two steps from L-tryptophan. A mixture of L-tryptophan (10 g, 0.05 mol), *t*-BuOH (19.5 g, 0.26 mol), and trifluoroacetic acid (75 mL) was stirred for 1 h. The volume was reduced, and the solution was triturated by 5% NaHCO_3_. The product was dried to provide a 60% yield of L-2,5,7-tri-*tert*-butyl-tryptophan. The Boc-protecting group was attached using di-*tert*-butyldicarbonate in dioxane.

The peptides **105**, **109**, and **110** were all prepared using consecutive solution phase peptide couplings according to the general method starting from isopropylamine, 2-phenethylamine, and *n*-hexylamine, respectively. Peptide **108** was prepared similarly starting from l-arginine methyl ester. Peptide **107** was prepared in the same manner, starting from 2-phenethylamine, but using Boc-l-Phe­[4-(2-naphthyl)]-OH instead of Boc-L-2,5,7-tri*tert*-butyl-tryptophan-OH.

### Antimicrobial Activity

#### Determination of Minimum Inhibitory Concentration (MIC)

MICs were determined using the microbroth dilution method for antimicrobial susceptibility testing published by the Clinical and Laboratory Standards Institute (CLSI, formerly NCCLS).^46^

MIC estimations were performed using wet plates containing the antibacterials. Following normal practice, all the plates containing Mueller–Hinton broth were prepared in advance, frozen at −70 °C on the day of preparation, and defrosted on the day of use. Susceptibility to comparator agents was determined using current CLSI breakpoints.

#### In Vivo Kill Kinetics Measurements

Fresh overnight colonies from a 5% Horse Blood Agar plate were suspended and diluted in 0.9% saline to approximately 1 × 10^8^ CFU/mL. A total of 50 μL of bacterial suspension was added to 9.95 mL of Mueller–Hinton broth and incubated at 35 °C with gently shaking. After 1 h of incubation, 0.5 mL of broth was replaced with 0.5 mL solution of peptide **109**, vancomycin, or dicloxacillin. Mueller–Hinton broth was used in the control groups. Samples for colony determination were taken at time 0, 10, 30 min, 1, 2, and 5 h after addition of test solutions. Each sample was diluted in saline with Triton X, and 20-μL spots were applied on 5% Horse Blood Agar plates. Immediately after sampling, 100 μL of nondiluted sample was spread on an agar plate to determine colony counts. All agar plates were incubated 18–22 h at 35 °C in ambient air.

#### Determination of Spontaneous Resistance

The frequency of the occurrence of bacterial colonies showing resistance was determined as a function of the total viable bacterial population. Selection was made on plates containing 2, 4, and 8 times the determined MIC for a particular strain using 1 mL of concentrated suspensions of bacteria at approximately 10^10^ per mL. After incubation overnight at 35 °C, growth was observed. When confluent growth occurred, a random selection was taken from the agar plate and used to reinoculate agar containing antimicrobial at the same concentration as the original selection. Resistance was confirmed if growth occurred after subculturing and incubation overnight at 35 °C.

#### Selection and Amplification of Resistance during Passage at Subinhibitory Concentration

A series of 8 concentrations of each antimicrobial was investigated against each test isolate as a macrobroth dilution MIC determination. Growth from the highest concentration allowing heavy growth after 18–24 h incubation (i.e., 0.5 × MIC) was then taken and diluted 1:100 and the MIC test repeated (passage 1). This passage was repeated continuously for a further 13 passages (i.e., 14 passages in total). When an increase in MIC was observed, the range of concentrations tested was increased accordingly for the next passage.

Any isolates showing raised MIC were stored at −70 °C. At the end of the study, those isolates with raised MIC at passage 14 were retested to confirm stable resistance development.

#### *In Vivo* Pharmacokinetic Measurements

The pharmacokinetics of peptide **109** in mice was investigated following single dose administration of 15 mg/kg IV and 30 mg/kg SC. Plasma and urine were collected at 5, 15, 30, 60, 120, and 180 min after administration. At each time point, urine was collected from the mouse by gentle compression of the abdomen before the mouse was anaesthetized with CO_2_ and blood was collected from axillary cutdown. The blood was centrifuged at 2000 G for 10 min and plasma was collected. The urine and plasma samples were kept at dry ice during the study period. The concentration of **109** in plasma was determined by HPLC. Estimation of the elimination half-life (*t*_1/2_) was performed by noncompartmental analysis (NCA) using WinNonlin version 5.01 (Pharsight Corporation). The IV results were analyzed by NCA, bolus IV administration, and the SC results were analyzed by NCA, extravascular administration.

#### Ethical Considerations

All clinical studies were registered by ClinicalTrials.gov, and the registration numbers are given in Table 12. In general, all studies were approved by the competent authorities and performed under signed written informed consent document by patient, parent, legal guardian, or caretaker.

The preclinical animal studies were performed by GLP certified CROs in Canada, Denmark, or the UK. All studies were performed in accordance with the guidelines set out by the national/regional councils on animal care, and the procedures for the studies were approved by the local animal policy and welfare committees.

## Supplementary Material





## Notes

HPLC chromatograms of peptides **105**, **107**, **108**, **109**, and **110** (PDF)
Molecular formula strings and associated microbiological and cytotoxicity data for peptides **105**, **107**, **108**, **109**, and **110** (CSV)

## Abbreviations

3- and 1-letter codes: 3- and 1-letter codes for proteinogenic amino acids according to IUPAC IUB are used in the article. Non-proteinogenic amino acids are described with adapted 3-letter codes. Peptide **109** has over the years had different names that can be found in the literature. The names include Ltx 9, LTX-109, AMC-109, and voxvoganan. The names can be used interchangeably
AMP: antimicrobial peptide
ATCC: American Type Culture Collection
AATCC: American Association of Textile Chemists and Colorists
MIC: minimum inhibitory concentration
API: active pharmaceutical ingredient
MRSA: methicillin-resistant *Staphylococcus aureus*
MRSE: methicillin-resistant *Staphylococcus epidermidis*
PLA: poly­(lactic acid) or poly­(lactide)
PGA: poly­(glycolic acid)
PCL: polycaprolactone
PDO: polydioxanone
LSR: liquid silicone rubber
HCR: high consistency rubber
