# Supplementary material for: Discovery and Development of Voxvoganan: First-in-Class Synthetic Antimicrobial Peptidomimetic
Source: J Med Chem. 2026 Apr 20;69(9):9908–27. doi: 10.1021/acs.jmedchem.6c00102 (PMC13181764; doi:10.1021/acs.jmedchem.6c00102)
Supplement: Supplementary file 1 [file jm6c00102_si_001.pdf]

SUPPLEMENTARY INFORMATION

Discovery and development of Voxvoganan – a first-in-class synthetic antimicrobial peptidomimetic

Wenche Stensen[a,b], Dina Jurman[d], Jon Lind[c], Pontus Lundberg[c],
John S. M. Svendsen[a,b*]

[a]UiT The Arctic University of Norway, 9019 Tromsø, Norway
[b]Amicoat AS, Mølnholtet 42, 9414 Harstad, Norway
[c]Amicoat AS, Hagaløkkveien 26, 1383 Asker, Norway
[d]Pharma Holdings AS, Sjøgata 2, 9008 Tromsø, Norway

Author Information

Corresponding author

J.S.M.S.: Phone (+47 90095072); e-mail, john-sigurd.svendsen@uit.no/john-sigurd.svendsen@amicoat.com

Table of content

Figure S1. Reversed phase HPLC chromatogram of Peptide **105** S2
Figure S2. Reversed phase HPLC chromatogram of Peptide **107** S2
Figure S3. Reversed phase HPLC chromatogram of Peptide **108** S3
Figure S4. Reversed phase HPLC chromatogram of Peptide **109** S3
Figure S5. Reversed phase HPLC chromatogram of Peptide **110** S4

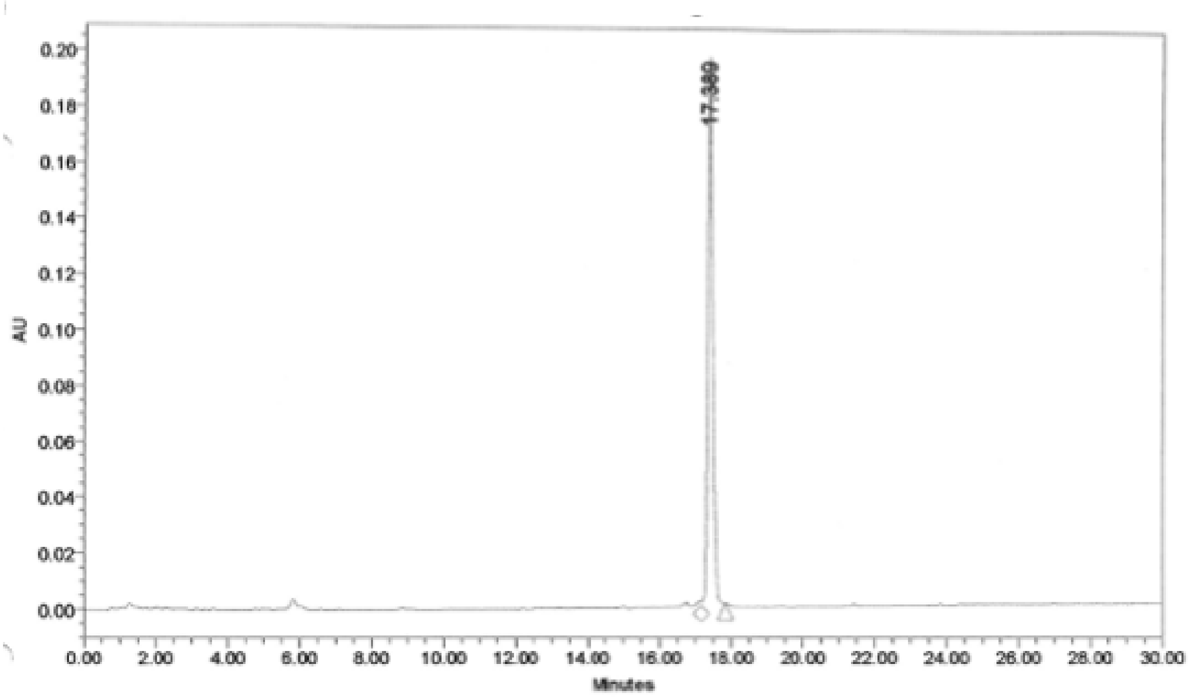

Peak Results

	RT	Area	Height	% Area
1	17.389	1959597	197397	100.00

Figure S1. Reversed phase HPLC chromatogram of Peptide **105**.

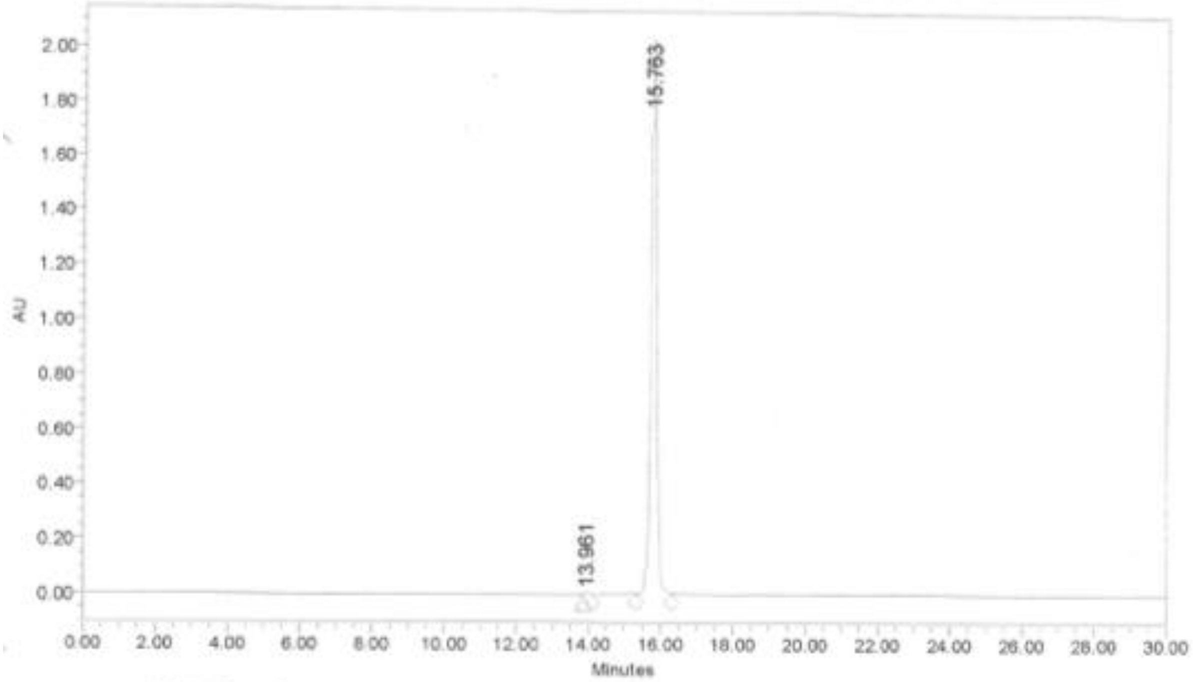

Peak Results

	RT	Area	Height	% Area
1	13.961	43716	8133	0.18
2	15.763	23862205	2041725	99.82

Figure S2. Reversed phase HPLC chromatogram of Peptide **107**.

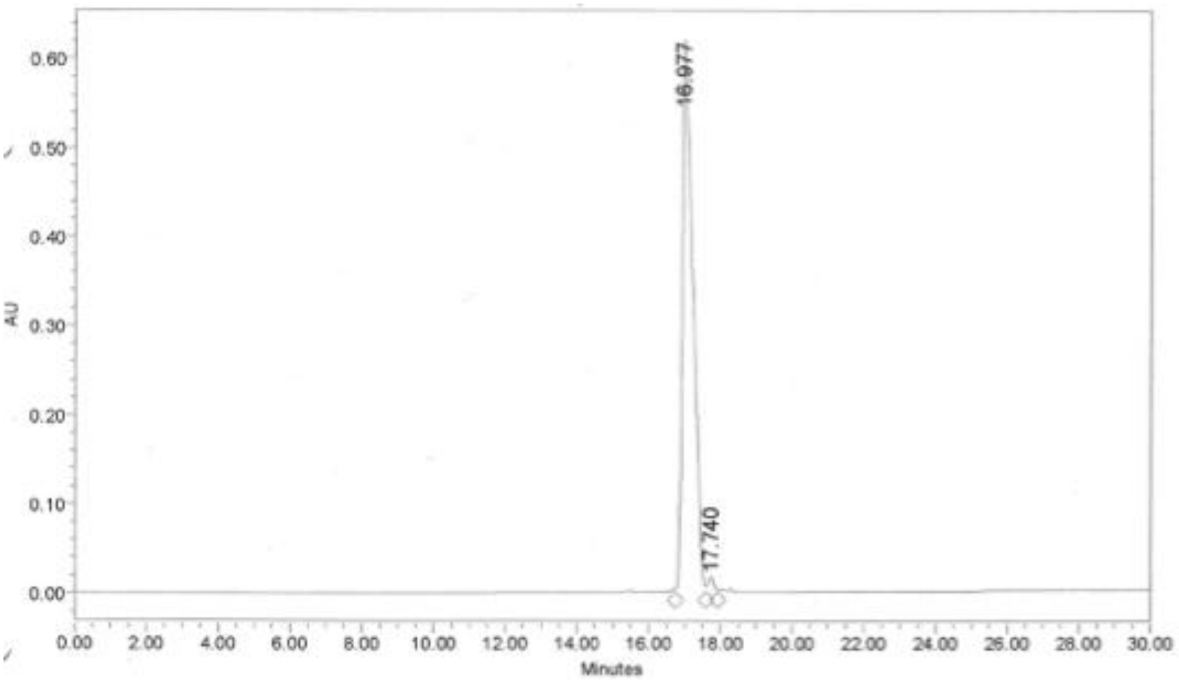

Peak Results

	RT	Area	Height	% Area
1	16.977	12906311	622103	98.57
2	17.740	186757	16961	1.43

Figure S3. Reversed phase HPLC chromatogram of Peptide **108**.

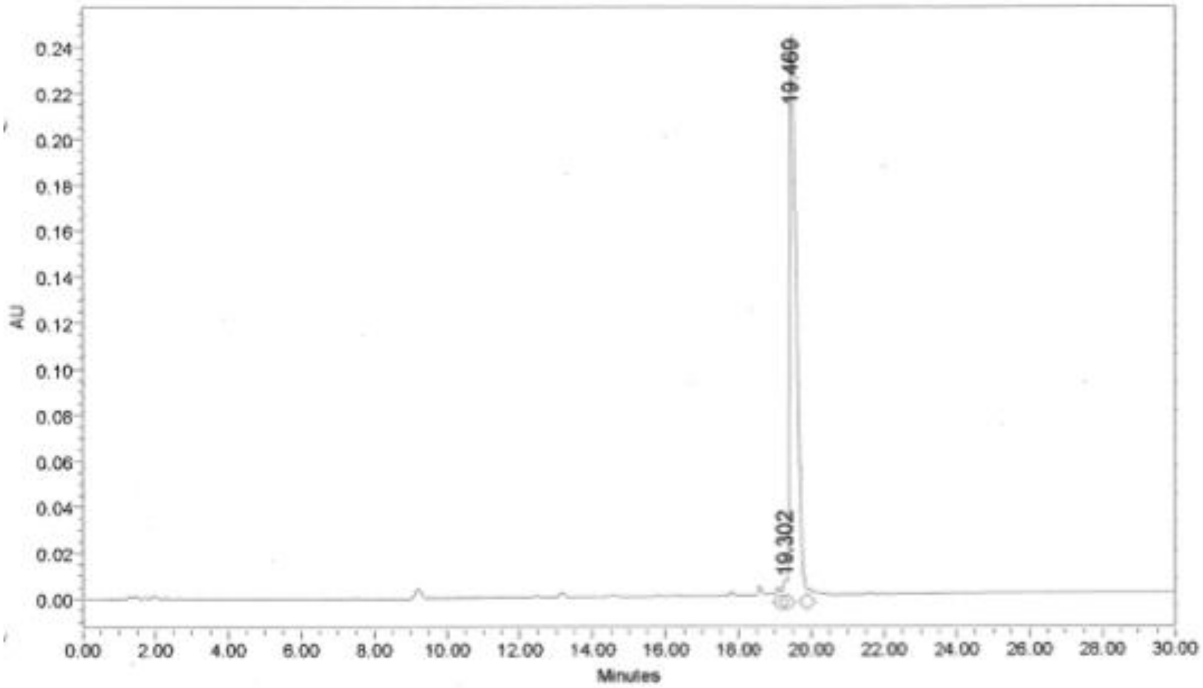

Peak Results

	RT	Area	Height	% Area
1	19.302	40872	5540	1.34
2	19.469	3002733	243533	98.66

Figure S4. Reversed phase HPLC chromatogram of Peptide **109**.

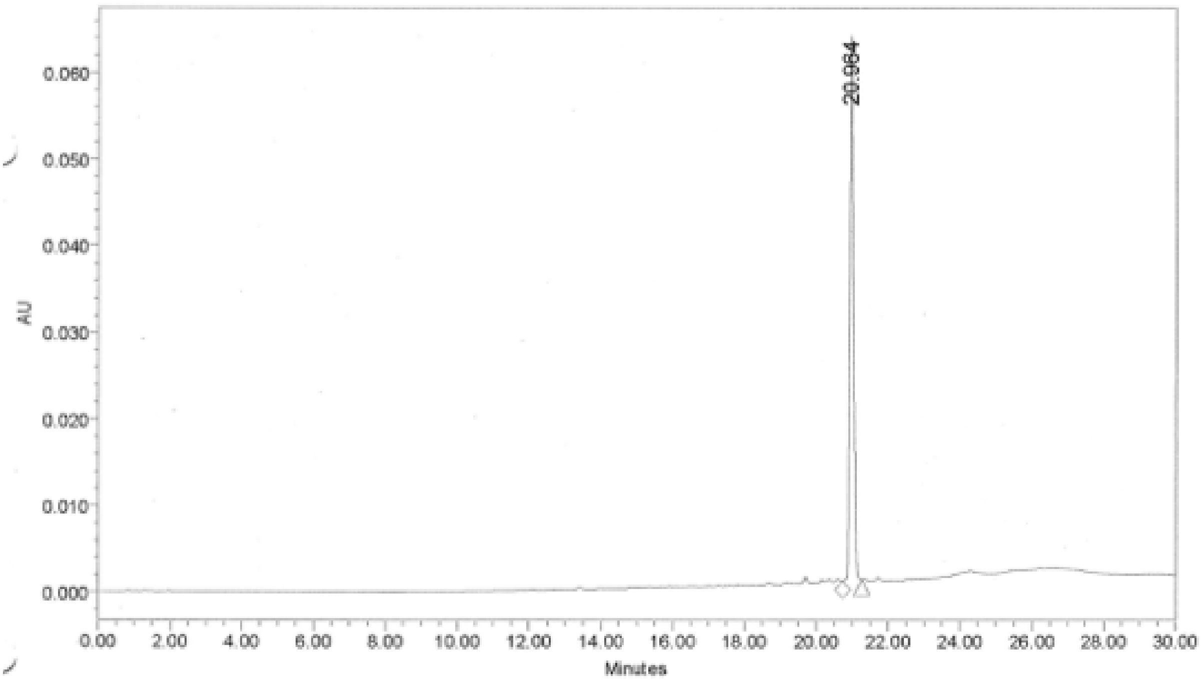

Peak Results

	RT	Area	Height	% Area
1	20.964	443949	62991	100.00

Figure S5. Reversed phase HPLC chromatogram of Peptide **110**.
